# ELI: A Conversational LLM-Based Interface for Human–AI Driving Teams and Its Impact on Performance and Driver Status

**DOI:** 10.3390/s26165228

**Published:** 2026-08-18

**Authors:** Evelyn Vasquez, Alanis Negroni, Juan Peña, Iyadunni Adenuga, Juan Medina-Lee

**Affiliations:** 1 Department of Electrical and Computer Engineering, Faculty of Engineering, University of Puerto Rico, Mayaguez Campus, Mayaguez, PR 00680, USA; evelyn.vasquez@upr.edu (E.V.); juan.pena3@upr.edu (J.P.); 2 Department of Computer Science and Engineering, Faculty of Engineering, University of Puerto Rico, Mayaguez Campus, Mayaguez, PR 00680, USA; alanis.negroni@upr.edu; 3 Department of Computer Science and Technology, The Dorothy and George Hennings College of Science, Mathematics and Technology, Kean University, Union, NJ 07083, USA; iadenuga@kean.edu

**Keywords:** LLM-based interface, human–AI driving teams, highly automated vehicles, driver monitoring system, human factors

## Abstract

Highly automated vehicles often rely on takeover requests (TORs) that lack contextual transparency, treat drivers as passive fallbacks, and lead to poor situational awareness. To address this challenge, this study presents the Empowering Language Interaction (ELI) framework, a conversational interface powered by a large language model that supports bidirectional negotiation and collaborative human–AI teamwork. Using the CARLA driving simulator, 28 participants compared ELI with a conventional TOR baseline in both urban and peri-urban driving scenarios. The study employed a multidimensional evaluation approach, integrating telemetry data on driving performance with continuous monitoring of physiological indicators (electrocardiogram and electrodermal activity) and subjective questionnaires to assess driver trust and engagement. Results indicated that ELI sustained continuous driver engagement and improved the subjective comprehension of the vehicle’s state. Physiologically, the conversational interface maintained active cognitive load, preventing the abrupt autonomic spikes characteristic of traditional takeover requests. Furthermore, ELI outperformed the TOR baseline in safety metrics by reducing out-of-lane events and maintaining greater safety margins. Conversational interaction has shown potential to transform drivers from passive supervisors into active teammates, improving joint decision-making without inducing over-reliance on the automated system.

## 1. Introduction

Highly automated vehicles are becoming more capable every year. Today, we even have robotaxis able to transport people without a human driver in some cities in the USA and China. Still, the operational design domain of these vehicles remains constrained to specific infrastructures, road conditions, good weather, stable internet connection, etc. So, what happens to the rest of the world? Are fully autonomous vehicles currently a feasible technology for the entire population? We do not believe so. Currently, some companies offer vehicles with automation levels 2 and 3 (conditional automation) [1], but this kind of automation may cause a reduction in the driver’s engagement, which increases the response time and the risk of incidents when regaining control of the vehicle [1,2].

This is especially relevant because the vehicle may operate autonomously under defined conditions but still require human input when the system reaches its operational limits [3,4,5]. Traditional interaction strategies, such as takeover requests, usually notify the driver when intervention is required, but they often provide limited information about why the situation is occurring, what the system has perceived, or which alternatives are available [6,7]. As a result, the driver may be asked to intervene without fully understanding the traffic context or the system’s reasoning.

Recent advances in artificial intelligence and large language models (LLMs) have created new opportunities for driver–vehicle communication. LLMs have been explored in automated driving for scene understanding, reasoning, planning, trajectory prediction, and explanation generation [8,9,10,11]. These capabilities suggest that natural language could be used not only for information, entertainment, or predefined commands but also as a communication layer through which the system explains its interpretation of the driving situation and the driver can provide information, ask questions, or express disagreement. Artificial intelligence has also made significant advances in vision-based models. They can be used for specific tasks, such as detecting AI-generated content [12], as well as more general activities, such as decision-making in autonomous vehicles, by using one of the promising technologies in automated vehicles: vision–language models. These models can reason and make decisions by analyzing the context in images. Even though they are rapidly improving, they still face several challenges, including hallucinations, high latency, and ethical considerations, particularly for vision-based systems [13].

In the context of advanced intelligent vehicles, human–machine collaboration extends beyond simple task allocation to dynamic, interdependent joint decision-making [14]. Modern multiagent teamwork requires entities to share objectives and mental states, actively coordinating cognition and skills while maintaining mutual predictability, directability, and common ground [15,16]. An efficient collaboration system must guarantee observability, allowing the human operator to understand the automation’s state and planning processes, and directability, enabling fluid redirection of the vehicle’s activities when intervention is required [15]. Recognizing that autonomy must respect team interdependency, an autonomous driving system (ADS) must be designed to foster mutual understanding through predictable, transparent task management [16]. In contrast to deterministic perception problems, where data from onboard sensors establishes a single objective reality, leaving no room for disagreement, tactical tasks such as lane changes and intersection crossing involve subjective preferences and strategic evaluations, making them suitable candidates for dynamic negotiation [17].

From a human–AI teaming perspective, this changes the interaction from a simple alert mechanism to a more collaborative process. Instead of treating the driver as a passive supervisor who reacts only when automation fails, the driver and the vehicle can be considered two agents that exchange information and contribute to the decision-making process [17,18,19]. In this context, the quality of the interaction depends not only on vehicle performance but also on how the driver perceives the system’s ability to interpret traffic situations, how reliable it feels, and how the interaction impacts the driving decisions [20,21,22].

### 1.1. Similar Studies

Research on highly automated driving has extensively examined the challenges that arise when drivers are no longer continuously controlling the vehicle but may still need to intervene. A considerable part of this research has focused on TOR, which is commonly used in conditional automation to transfer control back to the human driver [4,5]. These studies show that takeover performance is affected by driver disengagement, secondary tasks, traffic complexity, and the time available before intervention. They also indicate that a fast response does not necessarily represent an adequate response, since the driver may take control without fully understanding the surrounding traffic situation or the reason for the request [4,23,24].

To reduce the limitations of interaction based only on control transfer, cooperative automation and human–machine interfaces (HMIs) have been developed to keep drivers informed and involved before a takeover becomes necessary. These systems use visual, auditory, haptic, and augmented reality elements to communicate detected objects, automation states, planned trajectories, and possible maneuvers [6,7,19]. For instance, cooperative interfaces have employed force-feedback steering wheels to signal a disagreement between the driver’s input and the automated system, while visual interfaces have displayed detected obstacles or alternative trajectories so that the driver can compare the system’s interpretation with their own [6,19]. These approaches support observability and allow the driver to participate without immediately assuming complete manual control.

Other cooperative HMIs have extended this participation to specific tactical decisions. Muslim et al. proposed an interface that allows drivers to approve or veto an automated lane-change proposal within a limited time window [25]. If the driver did not intervene within the available time, the system continued with the planned maneuver. This type of interaction demonstrates that driver participation can be introduced before maneuver execution and that the absence of a response can be treated as implicit approval. However, the interaction remains based on predefined controls and centers on the approval or rejection of a specific maneuver, which limits the driver’s ability to provide contextual information, formulate alternative requests, or express counterproposals in natural language.

Recent work has also examined conversational and explanation-based interaction inside automated vehicles. Conversational agents have been studied as mechanisms for improving user experience, acceptance, and trust during autonomous driving [26,27]. On the other hand, explanation-based HMIs have also been used to communicate why the vehicle made a decision or how it interpreted the traffic environment, with the objective of supporting trust and situation awareness [28,29]. In parallel, LLMs have been introduced into autonomous driving research for perception, planning, reasoning, explanation, and decision-making tasks [9,10,11,30,31,32]. Some studies have moved beyond internal vehicle reasoning and have connected natural-language instructions with driving behavior. For example, the Drive as You Say approach uses an LLM to interpret human instructions and adapt tactical driving decisions according to the requested behavior [30]. These studies indicate that conversation and explanations can increase the transparency of the automation and that simple language can be converted into commands that influence vehicle operation. However, following a spoken instruction does not, by itself, constitute negotiation, because the interaction does not inherently involve a proposal from the ADS, room for disagreement, a counterproposal, or a defined process for resolving conflicts between the human’s request and the system’s safety judgment.

Fang et al. introduced a framework that uses an LLM for reasoning and an external HMI to enable bidirectional communication between autonomous vehicles (AVs) and human-driven vehicles (HVs) [33]. The framework addresses interaction between different vehicles by combining reasoning, interaction memory, and feasible action retrieval. This work provides evidence that LLMs can support explicit communication linked to driving decisions. However, its interaction is oriented toward external AV-HV coordination rather than toward negotiation between a human driver and the ADS operating within the same vehicle.

Physiological measurements have also been used to examine drivers’ states during interactions with automated systems. Electrocardiogram (ECG) and electrodermal activity (EDA) data can provide information related to arousal, workload, stress, and cognitive or emotional responses [6,22,34]. These measurements complement questionnaires and driving performance by describing how the driver reacts during specific traffic events. Yi et al. showed that combining physiological, behavioral, interaction, and self-reported measures provided a more reliable representation of driver trust than relying only on subjective reports [22]. Similarly, Ayoub et al. demonstrated that physiological and eye-tracking measures could be used to predict changes in driver trust in real time under normal operation, false alarms, and missed-event conditions [34]. Morra et al. found that an HMI providing more information about the vehicle’s perception and planning reduced the reported stress of the experience, although it also increased cognitive load [6].

Overall, previous studies show a progressive expansion of the driver’s role in automated driving. Cooperative HMIs have enabled drivers to observe, approve, reject, or influence specific vehicle actions; conversational agents have supported more natural interaction; interfaces based on explanation have improved the communication of vehicle perceptions and decisions; and approaches using LLMs have connected human language with driving reasoning and behavior. Together, these studies provide the main foundations for developing driver-ADS interaction beyond conventional alerts and control transfer mechanisms, combining communication, transparency, driver participation, and support for tactical decision-making.

### 1.2. Research Gap

Although the related literature provides complementary mechanisms for communication, transparency, driver participation, and tactical decision support, these capabilities are generally implemented as separate interaction mechanisms [25,26,27,29,30]. Approval-based interfaces rely on predefined responses; conversational and explanation-based systems mainly support communication and transparency; and instruction-following approaches connect human requests with vehicle behavior without necessarily establishing a structured joint decision process between the human and the ADS. This separation limits the integration of natural-language interaction with the mechanisms for negotiating and resolving tactical maneuvers.

There has been little focus on combining unconstrained driver voice input with a time-limited negotiation procedure as part of the vehicle’s tactical decision-making architecture. Such interaction requires more than explaining a maneuver or executing a verbal instruction. It requires a structured mechanism through which either the driver or the ADS can initiate a proposal, provide contextual information, express disagreement, formulate an alternative request, and resolve the decision within the scenario’s temporal and safety constraints. The ADS must also retain the ability to reject unsafe requests before they affect maneuver execution. At the same time, there is limited evidence on how this form of negotiation affects driver engagement, situational awareness, calibrated trust, physiological responses, and vehicle comfort and safety within a single experimental evaluation.

### 1.3. Approach and Contributions

To address this gap, the main contribution of this work is the ELI system, a conversational interface that integrates natural-language communication with the tactical decision architecture of an autonomous driving system. While prior cooperative automation frameworks rely on visual or haptic feedback, and explanation-oriented interfaces function primarily as one-way information channels, this work introduces a joint decision-making strategy built on a bidirectional negotiation protocol. Either the driver or the automated system can initiate maneuver proposals related to lane changes, overtaking, stopping, advancing, or intersection traversal. The protocol defines how the driving team handles these proposals through agreement, disagreement, extra requests, or rejection based on safety. To ensure operational security, the system uses a time-limited confirmation window that adapts to the specific driving scenario, balancing the opportunity for driver participation with the time required to execute the maneuver.

To support this negotiation process, the second main contribution is a communication layer powered by an LLM. Going beyond simple system integration or open-ended conversational assistants, this architecture successfully links free-form human utterances with arbitrary data from the vehicle maneuver planner. It combines a visual interface, an auditory listener, and speech synthesis to allow communication in both directions. The language model acts as a semantic bridge, mapping the spoken input from the driver into constrained maneuver or perception messages that the vehicle’s maneuver planning stack can process safely. At the same time, when the automated system initiates an interaction, the driving context is used to generate spoken, human-readable explanations for the driver. Crucially, the driver can provide contextual information, agree, disagree, or initiate another request without giving the language model direct control of the car. The autonomous system retains full responsibility for safety, maneuver selection, and physical execution.

Finally, the study contributes a triangulated multidimensional evaluation of this conversational interaction. The ELI system was tested against a nonverbal takeover baseline in a high-fidelity driving simulator. The evaluation combines subjective user perception, physiological signals synchronized with specific traffic events, interaction records, and objective vehicle data regarding safety and comfort. This comprehensive design allows for a clear examination of how conversational negotiation affects driver engagement, situational awareness, trust, physiological stress, and overall driving performance.

### 1.4. Hypotheses

This study is structured around four core hypotheses, designed to assess how ELI’s conversational capabilities affect the human driver’s role relative to a traditional, nonverbal takeover request baseline. Specifically, we aim to assess the system’s influence on sustained driver engagement, shared situational awareness, calibrated trust, driving performance, and overall operational safety. The evaluation is guided by the following hypotheses:

**Hypothesis** **1.**  *Drivers using ELI will be more engaged in the driving loop, even when the vehicle is in autonomous mode.*

**Hypothesis** **2.**  *The ELI interface will increase drivers’ perception of the automated system’s ability to logically interpret traffic scenarios and improve human drivers’ situational awareness of surrounding traffic and obstacles.*

**Hypothesis** **3.**  *The ELI mode will increase humans’ trust in the system, but it will not lead to overtrust due to constant interaction and awareness of the system’s limitations.*

**Hypothesis** **4.**  *The ELI mode will deliver better driving performance across physical comfort and safety metrics in the vehicle.*

### 1.5. Paper Organization

The remainder of this paper is organized as follows. Section 2 presents the study’s materials and methods. This section includes a general overview of the proposed approach, the description of the system architecture, the development of the driving experiments, the participant configuration, and the data acquisition process. Section 3 presents the experimental results, including the system latency and interaction metrics, as well as the driver trust evaluation and limitations. Section 4 discusses the main findings in relation to the proposed conversational interaction paradigm, trust calibration, driver–vehicle collaboration, and future work. Finally, Section 5 presents the concluding remarks.

## 2. Materials and Methods

This section describes the materials and methods used to develop ELI. First, the system’s architecture and pipeline are detailed. Then, the human–ADS negotiation protocol is depicted. The experimental setup is described last, including the hardware and software components used during the driving experiments, the simulator configuration, the physiological monitoring devices, and the participant description. This structure provides the basis for understanding how the system was implemented, how the experiments were conducted, and how the data used in the analysis were collected.

### 2.1. General Architecture

**Figure 1:**
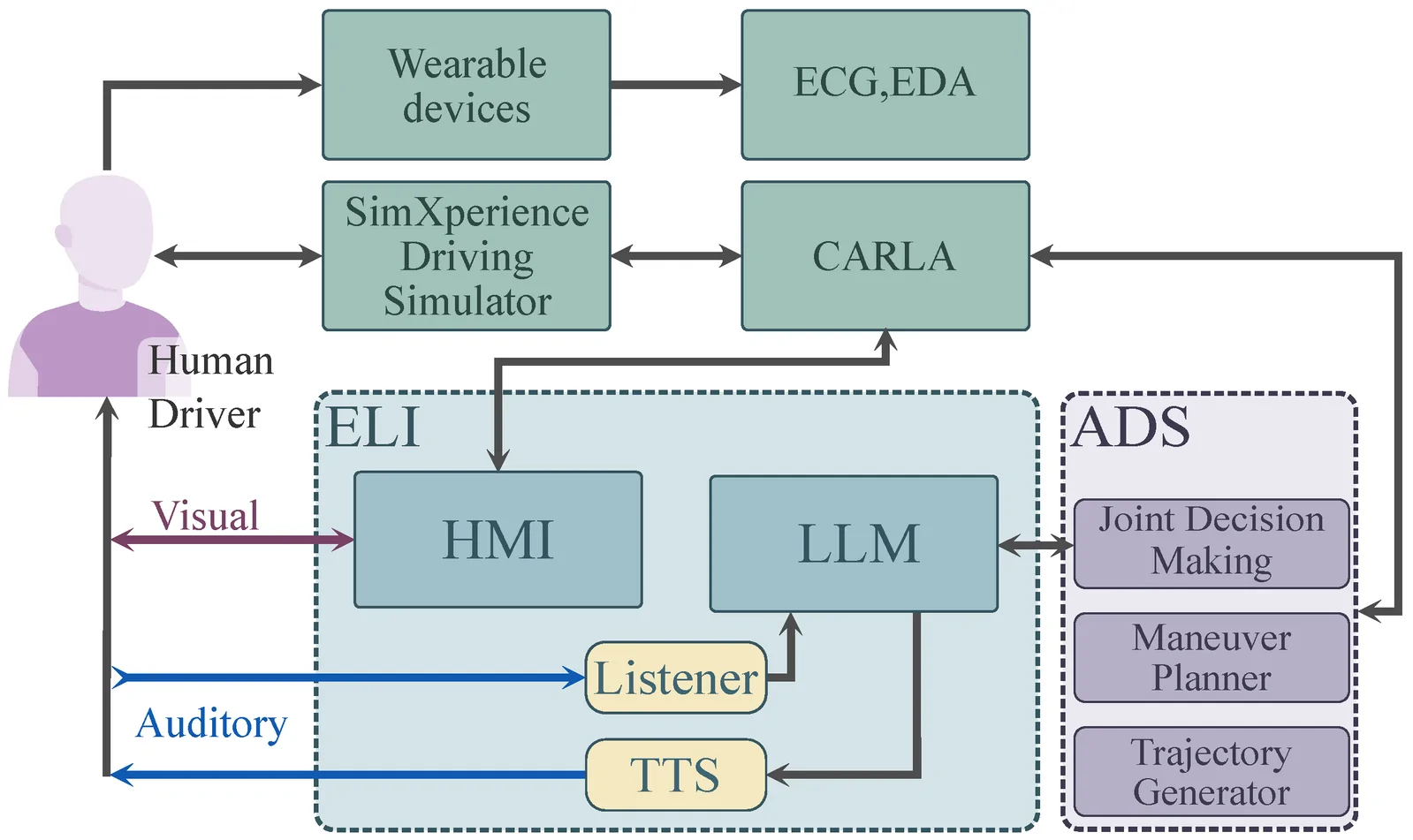
Team driving overview and architecture.

The core objective of ELI is to facilitate seamless communication between the human driver and the ADS. Figure 1 illustrates the architectural modules required to accomplish this. The upper section of the diagram depicts the driving simulator alongside the wearable devices employed to collect the driver’s physiological data. Structurally, the human driver is positioned on the left and the ADS on the right, with ELI acting as the central intermediary exchanging information with both agents. The human driver receives audiovisual feedback through a tablet-based human–machine interface and spoken responses from ELI. On the system side, ELI interfaces with the joint decision-making (JDM) module, a critical component of the ADS that manages the negotiation process with the driver. The JDM subsequently interacts with the maneuver planner to determine the strategic actions to be executed by the ego-vehicle.

The system’s operational workflow operates as a bidirectional loop. Auditory input from the human driver is captured by ELI, where an interpreter module identifies when the driver has finished talking, converts the audio to text data, and sends the message to a large language model, which decodes the specific intent, whether tactical (e.g., lane changes) or perceptual (e.g., obstacle identification). This decoded intent is transmitted as a structured payload to the JDM via the lightweight communications and marshaling (LCM) framework. Subsequently, the JDM evaluates the driver’s input against real-time telemetry from the CARLA simulator, including vehicle dynamics and the surrounding environment. The resulting system decision is sent back to ELI, synthesized into natural language, and delivered audibly to the driver via a text-to-speech (TTS) module. This exchange enables dynamic and fluid negotiation between the human and the automated driving system.

### 2.2. ELI Pipeline

ELI serves as the primary interface for direct interaction with drivers. Functioning as an HMI that prioritizes natural language, it is designed to emulate the dynamic communication between a driver and a human co-pilot. Instead of merely reacting to automated alarms, the driver can propose maneuvers or report environmental hazards in conversational language and subsequently receive spoken feedback that clarifies the vehicle’s actions and underlying reasoning.

**Figure 2:**
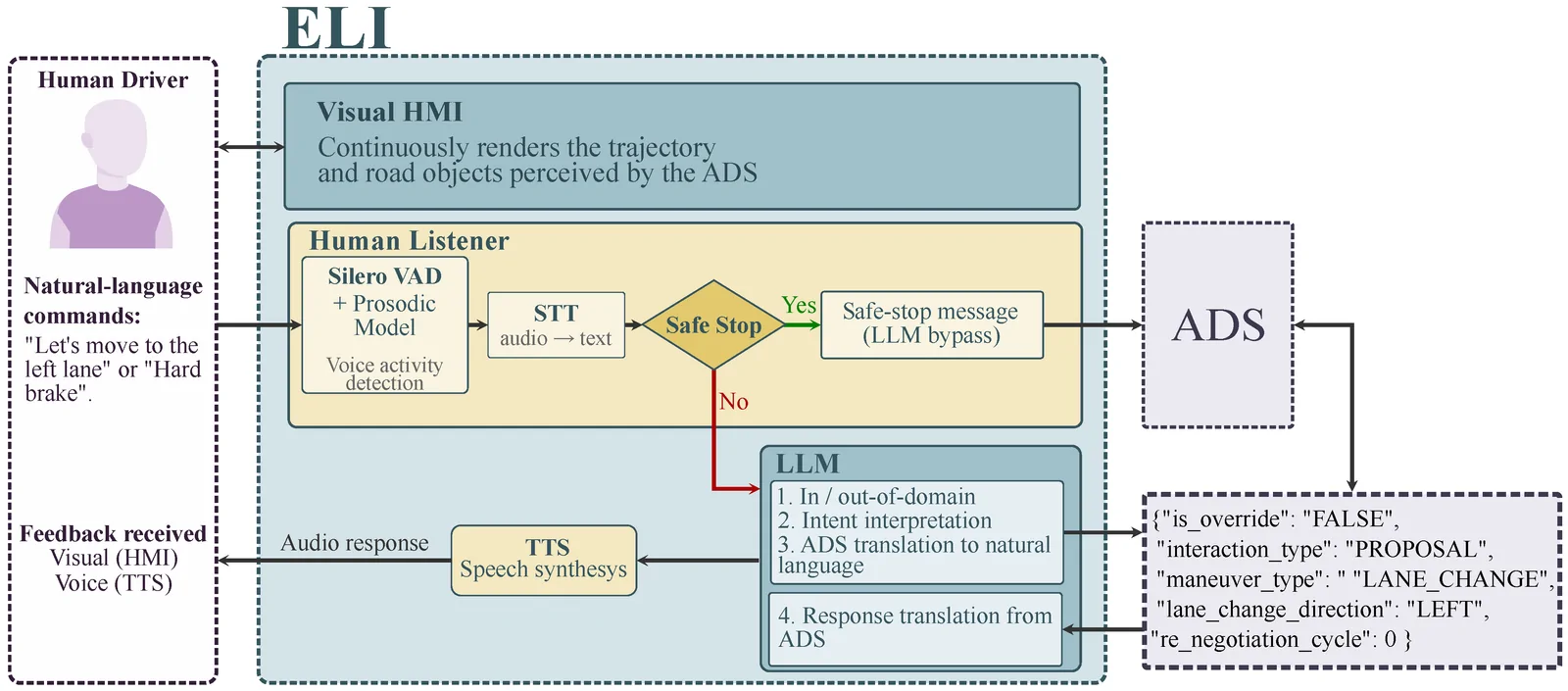
ELI pipeline and workflow.

Figure 2 illustrates the three core components of ELI: the visual HMI, the human–ADS interpreter, and the language model that facilitates natural communication. A detailed description of each module is provided below.

The front-end interface was developed as a Flutter [35] application running on a tablet adjacent to the driving station, while the backend processing is executed via Python greater or equal to 3.10 on a dedicated workstation. Flutter was selected for its cross-platform capabilities, allowing for rapid prototyping and seamless deployment across various devices from a single codebase. Finally, the interface integrates voice interaction with visual elements to maintain driver awareness and engagement.

#### 2.2.1. Visual HMI

**Figure 3:**
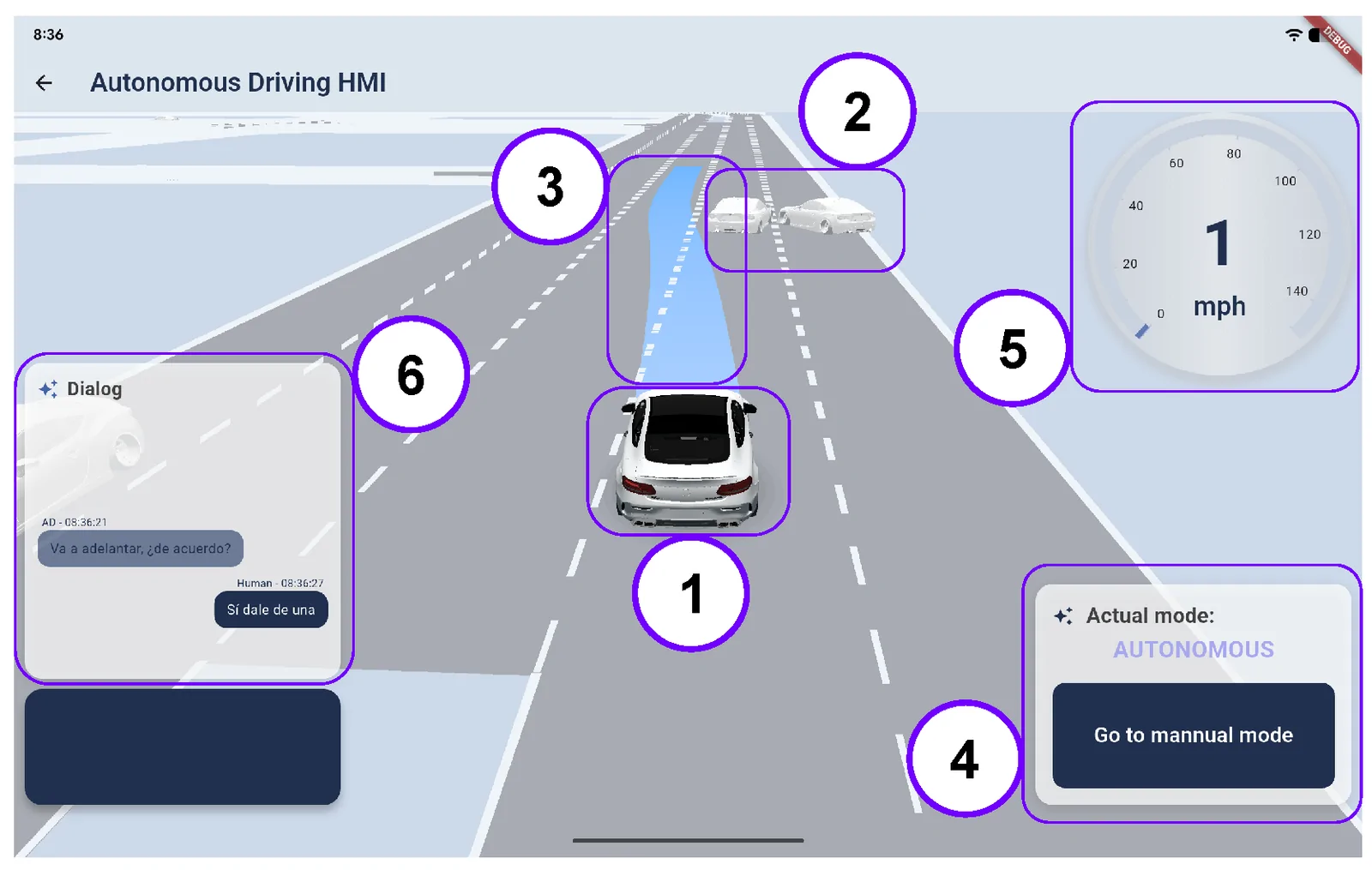
ELI interface layout showing the 3D driving scene, navigation map, vehicle status indicators, and conversation feed. (1) Ego-vehicle. (2) Detected obstacles. (3) ADS planned trajectory. (4) Control module. (5) Speedometer. (6) Conversation feed box.

The ELI interface was designed around the fundamental constraint that the user is actively monitoring or operating a vehicle; hence, it prioritizes voice as the primary interaction channel and uses visual elements strictly as complementary aids. To further accommodate the driver, the interface supports language selection (English and Spanish), allowing users to interact in their preferred language. The visual HMI was implemented on an Android tablet using Flutter. Figure 3 illustrates the functional zones of the HMI layout.

Central to the display is a 3D rendering of the CARLA environment, which shows the ego-vehicle (Element 1) and detected obstacles (Element 2), allowing the driver to instantly assess the system’s perceptual state. The interface also projects the automated system’s planned trajectory in light blue (Element 3), enabling the driver to anticipate upcoming maneuvers. The HMI also provides a continuous speedometer (Element 5). A dedicated control module (Element 4) indicates the active driving mode and features an override button for immediate manual intervention. This feature implies that the human driver may take control of the vehicle whenever they feel it is necessary. Finally, a conversation feed (Element 6) displays a rolling transcript of the driver-system dialogue, serving as a quick visual reference for recent verbal negotiations without relying on the driver’s memory.

#### 2.2.2. Human Listener

The ELI listener subsystem implements an always-on, streaming speech interface for the driver. Its main task is to identify when the human stops talking and deliver that message to the LLM. Audio is continuously acquired from the microphone using an input stream configured at 16 kHz with mono float32 sampling and then partitioned into short, fixed-length 30 ms frames. This frame-based representation serves as the common input to the downstream voice-activity and end-of-turn detection modules. The capture layer also maintains a bounded ring buffer of recent audio samples, providing a short pre-roll segment that can be prepended to an utterance upon speech onset detection. This design ensures that the system can preserve context immediately preceding the start of a command, which is especially important for short in-vehicle utterances.

Speech activity is detected in two sequential stages. First, a Silero voice activity detector (VAD) model [36] is applied to the incoming stream of frames to identify candidate speech segments. The VAD operates on fixed-size audio chunks and returns a probability of speech activity. Once speech is detected, the system begins accumulating the corresponding audio in an utterance buffer. A short silence interval acts only as a trigger for a subsequent turn-boundary assessment; it does not terminate the utterance by itself. This staged logic manages the inherent latency-versus-accuracy compromise in real-time voice interfaces: if the silence threshold is too short, speech gets split into too many segments, while a longer threshold creates perceptible delays before responses are generated.

The second-stage turn-boundary estimator is implemented using the pipecat-ai Smart Turn module, which integrates a wav2vec2 backbone, an attention-based pooling layer, and a lightweight classification head. This component inspects the buffered audio and estimates the probability that the speaker has finished their turn. Once this probability exceeds a specified threshold, the collected audio is released as a complete utterance for automatic speech recognition and interpretation. If the probability remains below the threshold, the silence is treated as a pause within an ongoing utterance, and the buffer is preserved. When the Smart Turn model is unavailable or cannot be loaded, the system reverts to a duration-based heuristic to maintain functionality in degraded conditions. Collectively, these mechanisms form a robust acoustic front end that enables continuous listening, turn-boundary detection, and low-latency speech capture in the driving environment.

In addition to the primary turn-segmentation pipeline, ELI includes a deterministic safety fast-path for urgent stop requests. After an utterance is segmented, it is transcribed using the speech-to-text (STT) module, and the system then evaluates the resulting text using a rule-based stop detector that matches a predefined bilingual vocabulary of emergency-stop triggers. When a stop trigger is detected, the utterance is converted directly into a canonical STOP maneuver message and forwarded to the downstream control stack without invoking the LLM-based intent classifier. This rule-based bypass is intended to minimize response latency and to ensure that high-priority safety commands are handled reliably and immediately.

#### 2.2.3. LLM-Based Communication

After speech transcription, the driver’s utterance is passed to the classifier module, which leverages an LLM to map the message into a constrained structured representation. The model is accessed via a remote MLX inference server. The wrapper in the Python service formats the request, forwards the prompt, and parses the model response into a JSON file. The LLM model used for this project is ‘gemma-3-4b-it-4bit’, a compressed, highly efficient version of Google’s Gemma 3 4B model designed to run locally on standard consumer hardware. This is the third generation of Google’s open-weight models, built on the same research as the Gemini family. In this study, the model serves as the inference backend for intent classification. This design separates the runtime service’s role from that of the model host: the application handles orchestration, schema construction, buffering, and post-processing, while the model server performs actual token generation.

The classifier does not prompt the LLM to classify the utterance in an unconstrained natural-language setting. Instead, the system constructs a domain-specific prompt, which asks the model to determine whether the message is relevant to driving. If the content is unrelated to the driving context, for example, background speech, chit-chat, music, or general conversational noise, the model is instructed to emit an out-of-domain signal. If the message is driving-related, the prompt then requires the model to distinguish between maneuver intent and perception reporting and to populate the appropriate structured fields. These fields include maneuver category, interaction type, urgency, and contextual metadata such as the negotiation cycle and safety criticality. The prompt is also language-aware, with Spanish and English example phrases used to guide lexical expectations while preserving the same enum semantics and schema constraints.

To reduce decoding variability and enforce validity, the classifier supplies a JSON schema over the output space. This schema defines a union of three mutually exclusive branches: out-of-domain, maneuver, and perception. The ‘oneOf’ formulation ensures that the model commits to a single branch of the schema at inference time, allowing the system to obtain a single-pass, structured prediction without an additional classification pass. The output is then post-processed to check for malformed or missing JSON; if found, it is treated as an out-of-domain fallback.

This approach yields a constrained language-model pipeline in which the acoustic front-end extracts a candidate utterance, and the LLM acts as a schema-constrained semantic interpreter. In effect, ELI maps spoken driver input onto a typed, machine-readable representation of intent while applying conservative rejection rules to non-driving or ambiguous speech. The result is a dialogue interface that remains responsive in real time yet is bounded by a well-defined control vocabulary suitable for high-level vehicle interaction.

A key design goal of ELI is local, offline operation, avoiding any dependency on cloud services for the safety-relevant path. Speech transcription, performed by the Silero VAD and the STT model, and intent classification (the gemma-3-4b-it-4bit model served via a local server) run entirely on-device on consumer hardware, requiring no network access. The only component currently relying on an external service is text-to-speech, which is used for the audio feedback channel and is not on the command-generation path to the ADS. This component can be replaced by a local TTS engine, making the entire pipeline fully self-contained and operable without any cloud connectivity. All model inference runs with deterministic decoding (temperature = 0.0), ensuring reproducible behavior independent of network conditions.

### 2.3. Negotiation Protocol

Traditional interaction strategies in conditionally automated vehicles, such as TORs, treat the driver as a passive supervisor and serve primarily as a unidirectional emergency fallback. These alerts often provide limited context, forcing the driver to intervene without a complete understanding of the system’s reasoning or the surrounding traffic environment. To address this limitation, a dynamic, bidirectional negotiation protocol was designed and implemented. The main goal of this protocol is to promote fluent human–machine collaboration by enabling the driver and the ADS to exchange information, discuss tactical maneuvers (such as lane changes or intersection traversals), and reach mutual agreements within safe time limits. By allowing the driver to ask questions, providing contextual input, and expressing subjective preferences before an action is executed, the negotiation protocol transforms the driver from a passive observer into an active co-pilot, which is the study’s main goal.

The negotiation protocol proposed in this study is built upon a preexisting use-case-independent autonomous driving architecture designed for complex urban environments [37]. The baseline system comprises a tactical maneuver planner [38] and a continuous trajectory generator [39]. The maneuver planner is responsible for high-level decisions such as lane selection and traffic regulation handling. Simultaneously, the trajectory generator dynamically computes a reachability set of feasible path candidates and associated speed profiles. The optimal trajectory is selected via a merit function that balances longitudinal and lateral comfort, safety, and utility. By evaluating a set of reachable trajectories rather than a single optimal path, the baseline system provides the flexibility needed to adapt to dynamic scenarios, forming the foundation for the human–ADS negotiation protocols introduced in this study.

#### 2.3.1. Joint Decision-Making and Negotiation Protocol

To implement this theoretical framework, the ADS was modified to incorporate a real-time negotiation protocol. Either the human driver or the ADS can initiate a maneuver proposal (e.g., perform a lane change, merge into an intersection, yield at the stop line, or perform a safe stop). Because driving scenarios are time-sensitive, negotiations cannot continue indefinitely, and the vehicle cannot hold for a long time while awaiting consensus. Therefore, the process begins with the ADS computing a time budget and starting a timer to govern the decision-making window. When the ADS proposes a maneuver, it sends the driving context to an LLM to generate a human-readable explanation, facilitating informed input. In the current implementation, we selected two types of collaborative maneuvers for the negotiation protocol: lane changes and merging into intersections. Each of those strategies is described below.

To manage these interactions, the protocol enforces strict temporal and cyclical limits. Under the implicit agreement via a timeout condition, the human is granted a defined time window to disagree or counterpropose. If the human does not provide input before the timeout condition is met, the system proceeds to execute the maneuver, effectively treating the timeout as an implicit agreement. If the human provides input, the timer stops, and the system evaluates whether they agree with the proposed maneuver.

**Figure 4:**
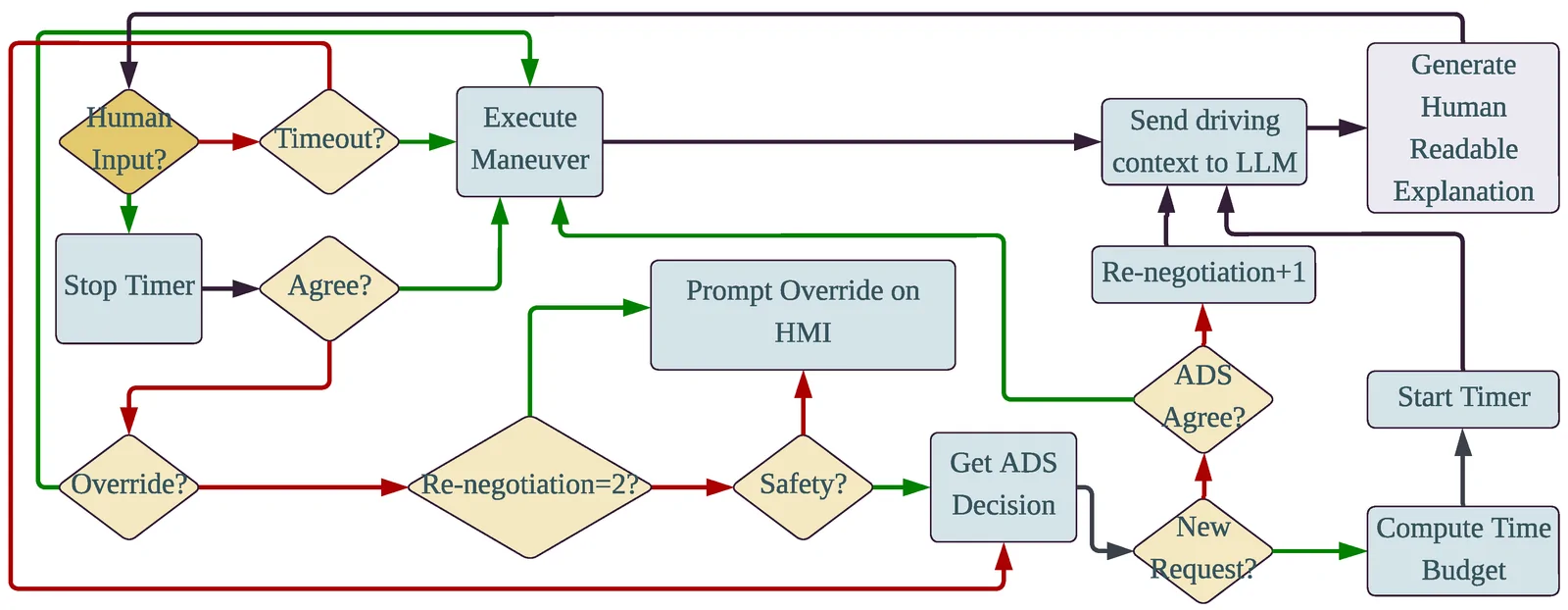
Negotiation protocol for ELI. Green arrows indicate a True value in the conditionals. Red Arrows indicate a False value.

Furthermore, the protocol employs cycle limits, ensuring that a single negotiation does not exceed two counterproposal cycles. If a disagreement persists, the system checks if the renegotiation limit has been reached. If this limit is hit, the human is prompted via the HMI to issue a forced override. Finally, regarding criticality overrides, if the ADS receives a new request or disagrees with a human proposal, it evaluates the situation’s safety criticality. If the ADS disagrees and the situation is deemed unsafe or highly critical, the system bypasses further negotiation and immediately prompts the human with a forced override option on the HMI. Figure 4 shows the complete flowchart of the implemented negotiation protocol.

#### 2.3.2. Time Budget Estimation

Time budget is one of the most critical aspects of decision-making in highly automated vehicles. Being able to make a decision before a critical state is reached is crucial to maintaining safety for all actors involved. To ensure a time-limited decision, this work proposes that the negotiation window is governed by a normalized urgency score, U∈[0,1]. This metric specifies how urgent it is for the ADS to perform a maneuver in order to avoid a safety-critical situation. If the urgency score reaches the safety level $U\geq S_{\mathrm{thd}}$ and there is no human input, the ADS makes a decision on its own.

The score is computed as the maximum of three normalized time-to-event metrics: the time-to-arrival to the closest regulatory element ($t_{\mathrm{reg}}$); the time remaining before the vehicle reaches the end of its current lane ($t_{\mathrm{lc}}$); and the remaining duration of the currently valid trajectory ($t_{\mathrm{traj}}$). The computing is performed as follows:

(1) $$U=\max(N\left(t_{\mathrm{reg}}\right),N\left(t_{\mathrm{lc}}\right),N\left(t_{\mathrm{traj}}\right))$$

where N(t) is a normalization function that defines urgency boundaries:

$$N\left(t,t_{\mathrm{safe}},t_{\mathrm{crit}}\right)=\left\{\begin{array}{cc} 1 & t\leq t_{\mathrm{crit}}\\ 0 & t\geq t_{\mathrm{safe}}\\ 1-\frac{t-t_{\mathrm{crit}}}{t_{\mathrm{safe}}-t_{\mathrm{crit}}} & \mathrm{otherwise} \end{array}\right.$$

where $t_{\mathrm{safe}}$ and $t_{\mathrm{crit}}$ are configuration parameters defining the minimum and maximum possible time budgets to perform a maneuver. The values of the parameters for the time budget computing are depicted in Table 1.

The critical time $t_{\mathrm{crit}}$ was selected based on the computational times of the ADS. It takes around 0.8 s to compute a new trajectory, so we selected a time limit that gives the planner enough time to compute the trajectory for the new maneuver. The safe time $t_{\mathrm{safe}}$ is set to match the time horizon of the ADS. This time horizon is multiplied by the ego-vehicle’s speed to determine the length of the current trajectory. Details of the trajectory generation process can be found in [40]. The safety threshold $S_{\mathrm{thd}}$ was initially set to 1, but it was reduced to 0.9 to make implicit decisions slightly faster. Note that reducing the value $S_{\mathrm{thd}}$ has a similar impact on the time budget as increasing $t_{\mathrm{crit}}$, but maintaining the two parameters allows us to change the normalization function in the future.

#### 2.3.3. Human-Augmented Lane Utility Function

When the human operator proposes a specific route or lane, the maneuver planner evaluates the request within the current negotiation cycle. The selection of the target lane $L^{*}$ is determined by maximizing a modified utility function *J* for each reachable lane *i*:

(2) $$J_{i}=J_{\mathrm{base},i}+\delta_{\mathrm{human},i}\cdot \omega_{\mathrm{pref}}$$

where $J_{\mathrm{base},i}$ represents the autonomous baseline utility derived from lane position, traffic density, and speed (more details about this baseline can be found in [38]); $\delta_{\mathrm{human},i}$ is an indicator function equating to 1 if the human has selected lane *i* via the human–machine Interface, and 0 otherwise; and $\omega_{\mathrm{pref}}$ is the internal weighting parameter, which was set to prioritize the human preference while still considering the rest of the variables that impact lane selection.

#### 2.3.4. Intersection Negotiation

To regulate automated transitions at intersections, the maneuver planner requires consensus before proceeding into potentially dangerous cross-traffic. When the autonomous system determines it is safe to cross, it proposes the merging maneuver to the driver and enters a temporary waiting phase.

During this phase, the system opens a dynamic confirmation window, bounded by the maximum time available before the vehicle reaches the stop line. If the driver explicitly agrees via the conversational interface, or if they do not intervene before the time window expires (implicit agreement), the system finalizes the decision. The speed planner then adjusts the vehicle’s trajectory to continue smoothly through the intersection without stopping. On the contrary, if the driver disagrees or requests a halt during the negotiation window, the transition is immediately rejected. The system’s target speed is set to 0 m/s, ensuring the vehicle comes to a complete and safe stop at the intersection boundary until the driver provides further instructions.

### 2.4. Experimental Setup

This section describes the complete experimental setup used in this study, including the simulation environment, wearable sensors, computational systems, participant group, data acquisition process, and experimental scenarios.

#### 2.4.1. Simulation Environment

**Figure 5:**
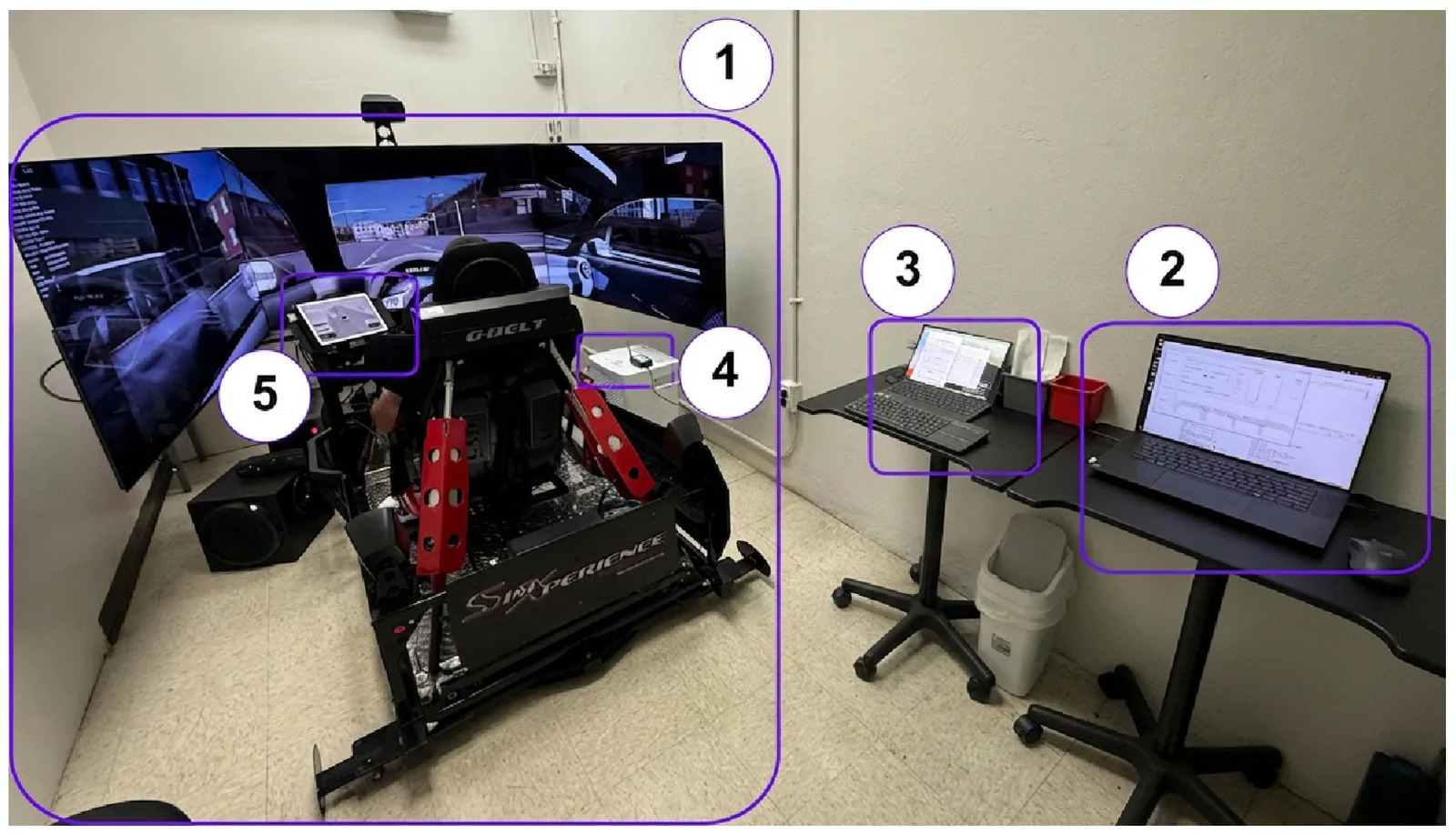
Driving simulator and experimental hardware. (1) Driving Simulator. (2) Computer for running the ADS. (3) Physiological data acquisition platform. (4) BIOPAC BioNomadix wireless receiver system. (5) Tablet running the HMI.

The simulation environment was built around the SimXperience Stage 5 platform (SimXperience, Akron, OH, USA) (item 1 in Figure 5), which served as the physical driving interface for the experiments. The platform provides motion cues through a multi-axis system and includes feedback from the steering wheel, seat, and audio system to reproduce common sensations associated with vehicle operation, such as longitudinal acceleration, braking, and lateral motion during turns. This configuration allowed the experiments to be conducted under repeatable conditions while preserving relevant physical feedback for participants. The driving simulation, including CARLA, was part of this setup (item 1), while the JDM module ran on a dedicated computer referred to as AI Driver (item 2).

Physiological signals were captured by a BIOPAC BioNomadix wireless system (BIOPAC Systems, Inc., Goleta, CA, USA) through a receiver (item 4) connected to a separate computer running AcqKnowledge data acquisition software v6.0 (item 3). A custom logging module was developed on top of AcqKnowledge to automatically tag each recording with the participant ID and experiment number, reducing the risk of mislabeled files during data collection. The ELI interface ran on a tablet mounted within the driver’s reach (item 5). A demonstration video of the system is available at https://www.youtube.com/watch?v=Xa7oBMYvrkg (accessed on 14 July 2026). Together, these elements provided the basis for collecting multimodal data during the interaction between the participant, the simulated vehicle, and the automated driving system.

The driving scenarios were implemented in CARLA 0.9.16 [41], an open-source simulation framework for autonomous driving research. CARLA provides configurable virtual environments in which vehicle behavior, traffic elements, and environmental conditions can be defined to meet the study’s requirements. In this study, CARLA was used to create controlled urban and peri-urban scenarios that allowed participants to interact with the automated driving mode under predefined events. CARLA cameras were used to reproduce the in-vehicle visual perspective and display the driving environment across the three screens of the SimXperience Stage 5 platform. These features enabled a consistent experimental structure across participants while collecting multimodal data on physiological responses and trust in the system.

#### 2.4.2. Participants

The experimental protocol was conducted with $N_{S}=28$ volunteer participants. Participants were recruited through a public call for participation distributed within the university community. Interested individuals contacted the research team and completed the informed consent process before participating in the study, in accordance with the corresponding institutional ethical procedures. Similar driving simulator studies have involved 28 participants to examine trust in automated vehicles through subjective evaluations, electrodermal activity, gaze, and driving behavior [42,43,44]. Studies combining subjective evaluations with ECG, electrodermal activity, and other physiological measures have also included between 31 and 34 participants [45,46].

The sample included participants with diverse demographic backgrounds, driving experience levels, and familiarity with driver assistance or autonomous vehicle systems. Specifically, 46.4% of the participants identified as female and 53.6% as male, as summarized in Table 2. Participants’ ages ranged from 18 to 46 years, with a mean age of 26.64 years and a standard deviation of 6.75 years, as shown in Table 3.

Participants’ driving experience ranged from 1 to 26 years, with a mean of 6.54 years and a median of 5 years. This variability enabled the study to include drivers with both limited and extensive driving experience, which is relevant to evaluating human interaction with assistance systems in autonomous driving scenarios. Regarding previous experience or knowledge of driver assistance systems or autonomous vehicles, 57.1% of participants reported no prior experience, 35.7% reported theoretical knowledge with little or no practical experience, and 7.1% reported practical experience using these systems. Therefore, most participants represented users with limited direct exposure to autonomous vehicle technologies, making the study suitable for evaluating how drivers interact with assistance systems under simulated autonomous driving conditions.

Since driver state may influence the interaction with assistance systems, participants also reported information related to their prior sleep time and stress levels. The average reported sleep duration was 6.43 h per night (SD = 0.84 h). Self-reported sleep quality during the previous week had a mean score of 3.43 out of 5, and reported stress level had a mean score of 3.21 out of 5. These variables provide additional context for interpreting participants’ behavioral and physiological responses during the experiments, as summarized in Table 3.

Across the experiment, all participants were exposed to the two assistance modes evaluated in this study, namely TOR and ELI, as well as two driving layouts: urban and peri-urban. Participants were randomly assigned to one of four experimental groups. The specific sequence followed by each group is presented in Table 4. These sequences formed a randomized, balanced, counterbalanced two-period crossover design, jointly balancing the order of the assistance modes and driving environments across the two experimental periods.

Each group included seven participants, corresponding to 25.0% of the total sample. As shown in Table 4, 14 participants experienced TOR-first and 14 experienced ELI-first, while the urban and peri-urban layouts were equally represented in the first and second experimental periods. This distribution reduced the systematic association of learning, adaptation, or fatigue with a particular assistance mode or experimental period. In addition, it prevented a specific assistance mode from being systematically associated with a single driving layout or position in the experimental sequence, thereby supporting a more reliable comparison of driver interaction across different autonomous driving assistance configurations.

#### 2.4.3. Data Acquisition

Physiological data were recorded using the BioNomadix system, a wireless wearable platform used for continuous physiological monitoring during the driving sessions. The system acquired the signals at a sampling rate of 2000 Hz, allowing high-resolution recording of the participant’s physiological responses throughout the experiments. In this study, electrocardiogram and electrodermal activity signals were collected as the main physiological data sources.

**Figure 6:**
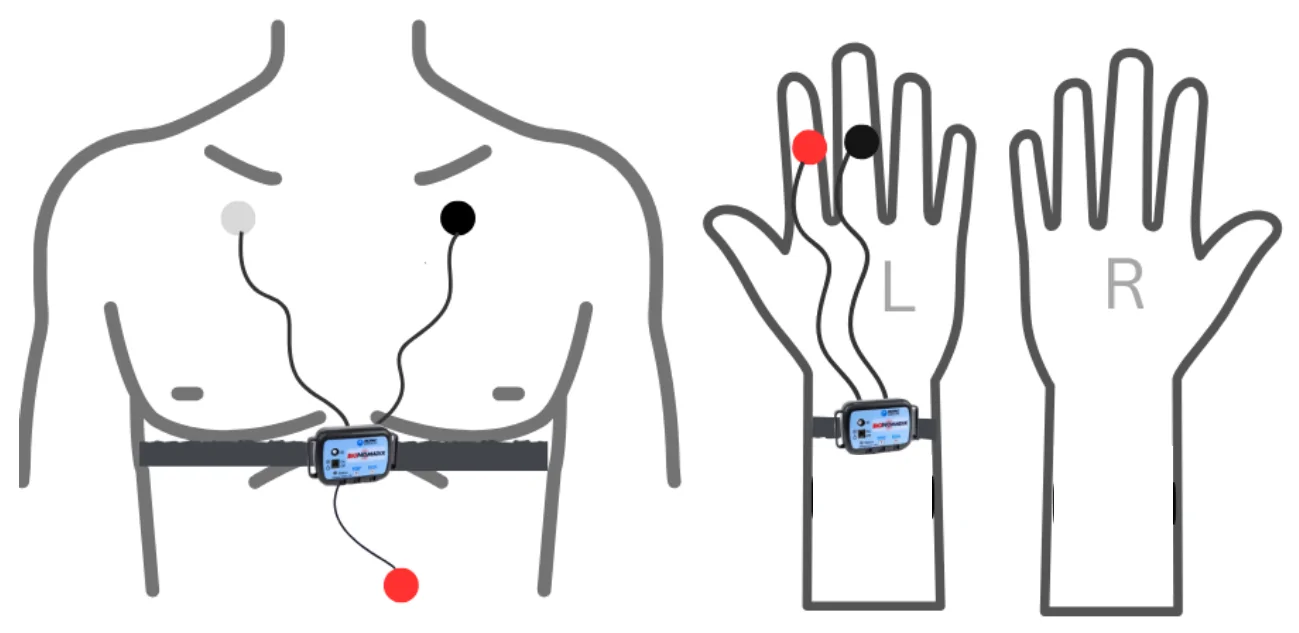
Location of physiological sensors on the subject’s body. The red, black, and white electrodes corresponded to the positive (E^+^), negative (E^−^), and ground (GND) inputs, respectively.

ECG signals were acquired with BioNomadix RSP&ECG sensors (BN-RSPEC-TGED-T), with electrodes placed on the participant’s chest in a Lead I configuration. EDA signals were recorded using a BioNomadix PPG&EDA sensor (BN-PPGED-T). For this measurement, electrodes were attached to the index and middle fingers of the participant’s left hand. Figure 6 shows the electrode placement on each subject.

As these devices use a wireless transmission protocol, some of the samples are lost during the transmission, sometimes due to electromagnetic interference, other times due to the subjects’ movement during the simulation. These sample losses are uncommon and may occur once every few seconds during an experiment. When they occur, the BioNomadix receptor provides a value of zero for each lost sample. To handle this loss of information, we process the data logs and apply an interpolation strategy whenever we encounter a zero value in the physiological data. We replace the zero values with linear interpolation between the preceding and succeeding non-zero values.

After acquisition and preprocessing, ECG and EDA signals were processed to extract the physiological features used in the analysis. The pipeline was implemented using NeuroKit2 [47], together with NumPy for numerical processing, and included signal cleaning, peak detection, and feature extraction over fixed 60-s sliding windows. For ECG signals, R-peaks were detected and used to compute heart rate variability (HRV) metrics describing the autonomic modulation of cardiac activity; specifically, the low-frequency to high-frequency power ratio (LF/HF) and the root mean square of successive differences (RMSSD) were retained for analysis. For EDA signals, the skin conductance level (SCL) and its temporal slope were extracted to characterize tonic electrodermal arousal.

To mitigate motion-related beat-detection errors on HRV (particularly relevant for RMSSD), ectopic and misdetected beats were corrected using the Kubios artifact correction method [48] prior to computing HRV metrics.

To enable within-subject comparisons across conditions, each feature was normalized relative to a per-session, per-subject baseline. Specifically, for each participant and each experimental session, the first 60 s of calm driving were used as the intra-session reference. Features with strictly positive distributions (mean SCL, LF/HF, RMSSD) were log-transformed prior to normalization, such that the reported values represent the log-ratio relative to the session baseline: $\Delta f=\ln f\left(t\right)-\ln{\tilde{f}}_{\mathrm{baseline}}$, where ${\tilde{f}}_{\mathrm{baseline}}$ denotes the median of the feature during the baseline window. Features that can take negative values (SCL slope) were normalized by simple subtraction: $\Delta f=f\left(t\right)-{\tilde{f}}_{\mathrm{baseline}}$. This approach removes inter-session tonic offsets while preserving within-session physiological dynamics, with Δf=0 indicating a response at the level of each participant’s own calm-driving baseline. Table 5 summarizes the full physiological feature set considered in this study.

#### 2.4.4. Driving Experiments

The driving experiments were designed to evaluate driver behavior and physiological responses under different assistance strategies in an automated driving context. In particular, this study compares two assistance modes implemented during autonomous vehicle operation: the collaborative assistance mode, ELI, and the baseline, a strategy based on takeover requests (TORs). Both modes were evaluated using simulated driving scenarios in CARLA, where participants were exposed to predefined traffic situations that could require a transition of control or increased driver attention.

Two driving experiments were conducted in different CARLA environments. The first experiment was conducted in an urban layout (CARLA’s Town03), while the second was conducted in a peri-urban layout (CARLA’s Town04). Each experiment consisted of a predefined route in which the ego-vehicle operated in autonomous mode and encountered critical driving situations. These situations were designed to evaluate how participants responded to events that could require manual intervention or increased awareness.

**Figure 7:**
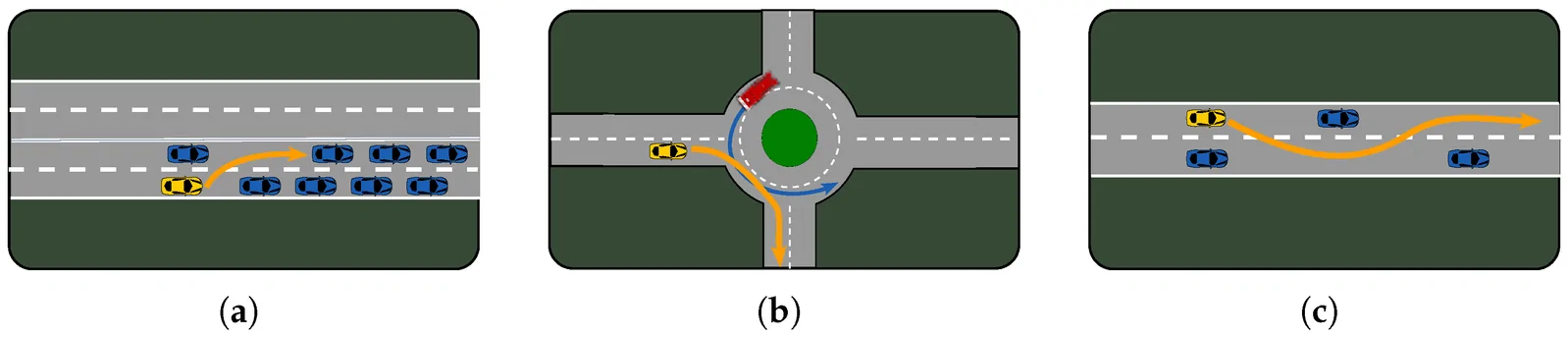
Use cases included in the urban layout. The Yellow line corresponds to the ego-vehicle’s expected path. (**a**) Urban use case 1. (**b**) Urban use case 2. (**c**) Urban use case 3.

**Figure 8:**
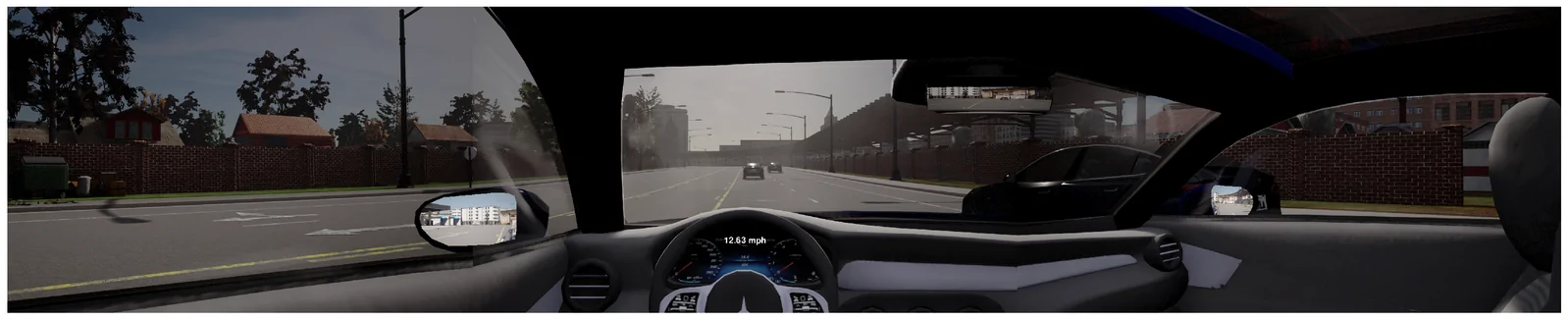
Simulator view of urban use case 3 in the urban layout.

The urban layout experiment consisted of an approximately eight-minute route with three use cases (UCs) in which the participant could interact with the ADS to handle the situation (for ELI assistance) or take control of the vehicle (for TOR assistance). These use cases are illustrated in Figure 7. The urban use case 1 (U−UC1) corresponds to a traffic congestion scenario in which vehicles in the left lane move faster than the ego-vehicle. Urban use case 2 (U−UC2) corresponds to a roundabout scenario in which a fire truck is located inside the roundabout but is not detected by the ego-vehicle because a stopped vehicle in the inner lane blocks the ego-vehicle’s perception of the traffic situation. This situation was designed to evaluate the participant’s response to a partially occluded traffic conflict in which the automated driving system may not properly interpret the surrounding environment. Finally, urban use case 3 (U−UC3) corresponds to an overtaking scenario in which the ego-vehicle is positioned between two vehicles and must respond to the surrounding traffic conditions. A detailed view of Use U−UC3 as presented in the simulator is shown in Figure 8.

**Figure 9:**
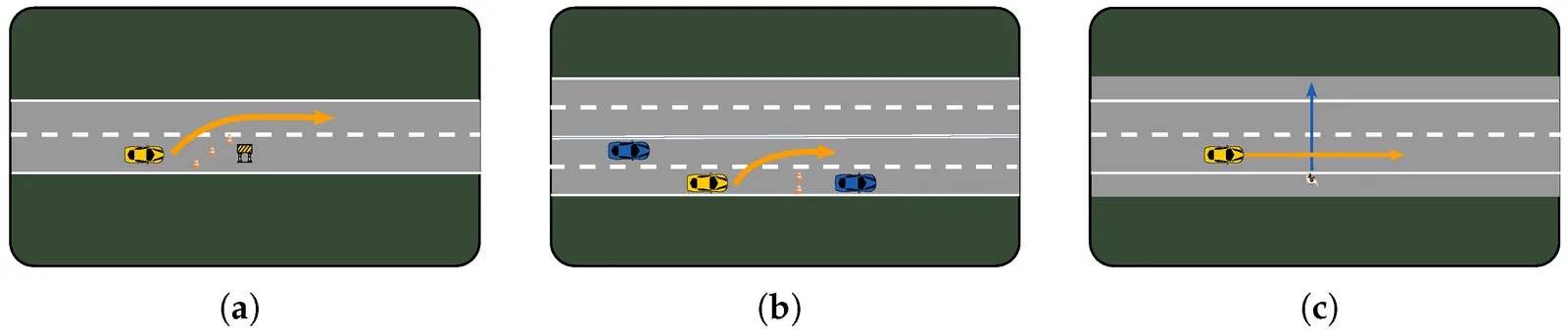
Use cases included in the peri-urban layout. The yellow line corresponds to the ego-vehicle’s expected path. (**a**) Peri-urban use case 1. (**b**) Peri-urban use case 2. (**c**) Peri-urban use case 3.

**Figure 10:**
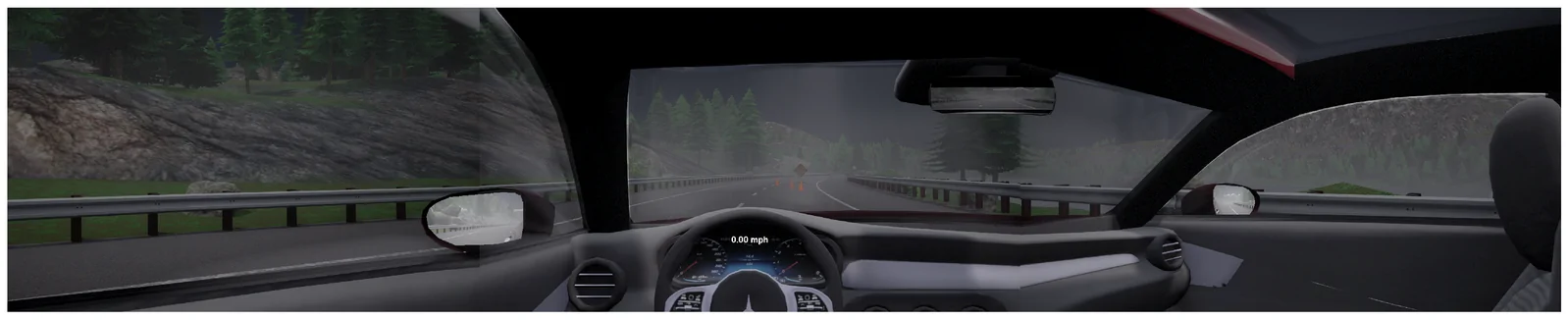
Simulator view of peri-urban use case 1 in the peri-urban layout.

The peri-urban layout experiment also comprised three predefined use cases, as shown in Figure 9. In the peri-urban use case 1 (P−UC1), an obstruction is present on the road, requiring the ego-vehicle to change lanes to continue along the route, as shown in Figure 10. In the peri-urban use case 2 (P−UC2), a vehicle experiencing a mechanical failure is stopped on the roadway, necessitating a lane-change maneuver. In peri-urban use case 3 (P−UC3), the ego-vehicle approaches and crosses an intersection while the traffic light for its lane is green. At that moment, a pedestrian crosses the road at an inappropriate location, outside the expected crossing area, and without the right of way. This creates a potentially unsafe situation, since the automated vehicle must detect the pedestrian and respond appropriately, while the participant may decide to intervene if they perceive that the system is not reacting in time.

#### 2.4.5. Experimental Protocol

**Figure 11:**
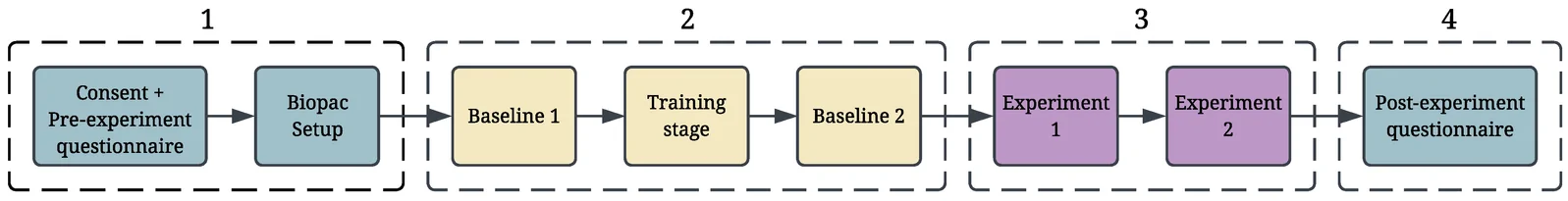
Experimental workflow. (1) Setup phase. (2) Training phase. (3) Experimental phase. (4) Post-experiment phase.

Figure 11 presents the protocol used to collect data from each participant, which was divided into four phases. In Phase 1, participants signed the informed consent form and completed a pre-experiment questionnaire covering demographic information and baseline self-reported characteristics. This phase concluded with the placement and configuration of the physiological sensors, as illustrated in Figure 6. Phase 2 began with Baseline 1, during which physiological data were recorded while the participant remained at rest. Participants then completed a training stage to familiarize themselves with the driving simulator and interact with the ELI assistance mode. This was followed by Baseline 2, in which physiological data were recorded while the participant drove manually. Each stage in Phase 2 lasted five minutes. In Phase 3, participants completed the two driving experiments according to the order and assistance conditions assigned to their corresponding group, as reported in Table 4. Finally, in Phase 4, participants completed a post-experiment questionnaire, which consisted of eight numerical items (four related to ELI and four related to TOR) and two open-ended questions aimed at assessing the performance and capabilities of each assistance mode. The specific content of the numerical items is detailed in Section 3.3, and the open-ended questions are described in Section 3.5.

## 3. Results

To evaluate the ELI system against the TOR baseline, we employed a multimodal evaluation strategy that captures both systemic and human-centric dynamics. The comparison of these assistance modes is structured across four key dimensions. First, we analyzed negotiation outcomes and driver response latencies to assess the conversational pipeline’s temporal efficiency. Second, we examined continuous physiological signals to quantify drivers’ cognitive load and stress responses. Third, we evaluated subjective driver perception and trust through quantitative surveys alongside a qualitative thematic analysis of user feedback. Finally, we assessed objective driving performance by comparing safety margins and physical comfort metrics derived from simulator telemetry.

### 3.1. Negotiation Outcomes and Driver Response Latency

To evaluate the collaborative efficiency of the ELI framework, we analyzed a total of 569 individual negotiation episodes collected across the 28 subject experiments. Some of these episodes were initiated by the human subject; others were initiated by the ADS. Each of the latter had been assigned a dynamically scaled confirmation window, W∈{3,4,5} s, during which the driver could accept, reject, or ignore the proposed maneuver.

**Figure 12:**
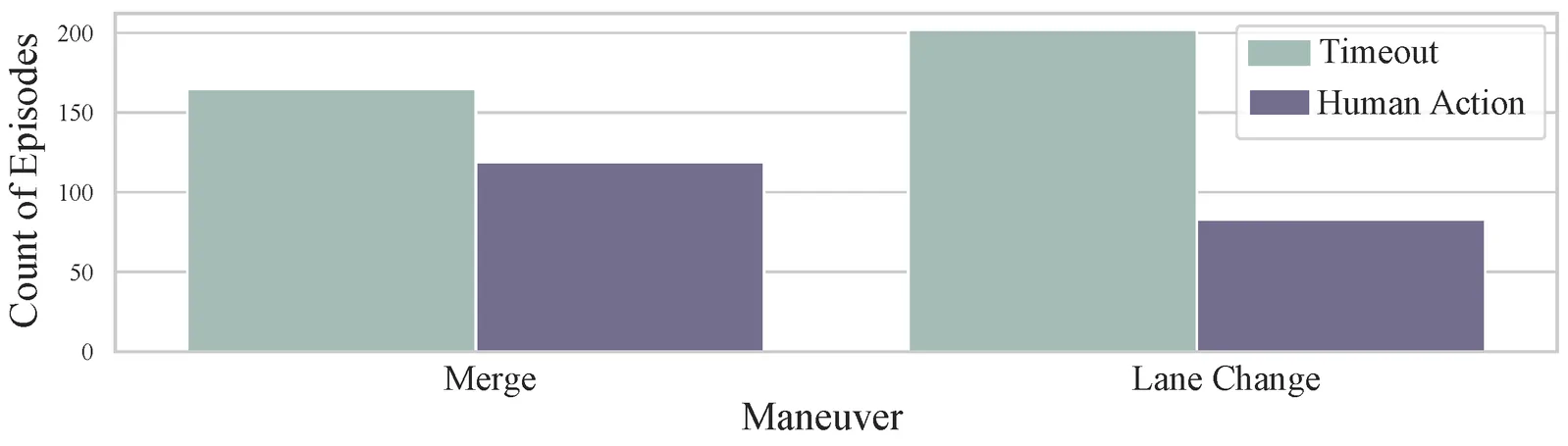
Distribution of negotiation outcomes by maneuver type.

The distribution of negotiation outcomes across maneuver types is depicted in Figure 12. For both lane change and merge proposals, the system resolved the majority of episodes via the ADS fallback after the confirmation window expired (70.9% and 58.1%, respectively), indicating that participants frequently allowed the planner to execute autonomously without intervening. These timeout episodes naturally reflected the expiration of the allocated window, with mean latencies of 4.20 s for lane changes and 4.53 s for merges. In contrast, human-resolved episodes represented 29.1% of lane changes and 41.9% of merges, suggesting that merge proposals elicited slightly greater driver engagement, possibly due to their higher perceived risk.

**Figure 13:**
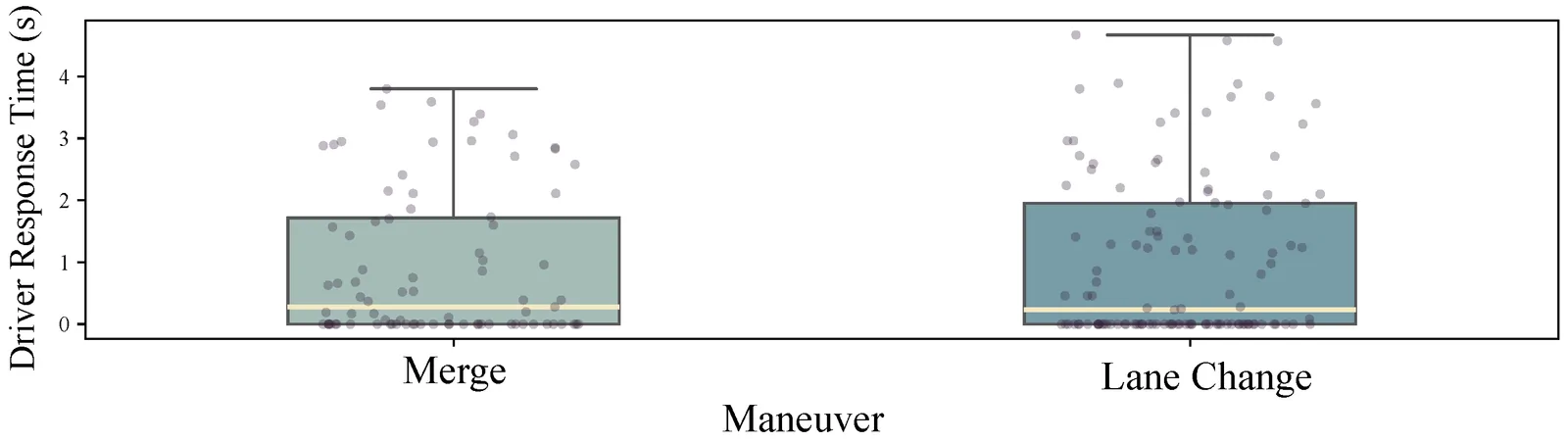
Driver response-time distribution for human-resolved negotiation episodes. The yellow solid lines represent the median values for each maneuver type, while the grey lines represent the minimum and maximum values.

Among the 202 human-resolved episodes, driver response latencies were notably fast, as illustrated in Figure 13. For lane-change maneuvers, drivers achieved a mean response latency of 0.94 s and a median response time of 0.28 s. Similarly, merge proposals yielded a mean response latency of 1.03 s and a median of 0.23 s. These rapid responses fell well within even the shortest available confirmation window of 3.0 s. Ultimately, this overall distribution confirms that when drivers chose to engage, the interaction was highly responsive and fully compatible with the latency constraints imposed by the dynamic time budget mechanism.

Table 6 summarizes the transitions to manual driving according to the experimental layout and the assistance mode used by the participants. For each assistance mode, the manual mode events column reports the total number of times participants took control of the vehicle, whereas the number of users in the manual mode column indicates the number of unique participants who entered manual mode at least once. Therefore, a participant could contribute more than one manual mode event in the same experiment. Overall, manual control was activated more frequently and by more participants in TOR mode than in ELI mode.

**Figure 14:**
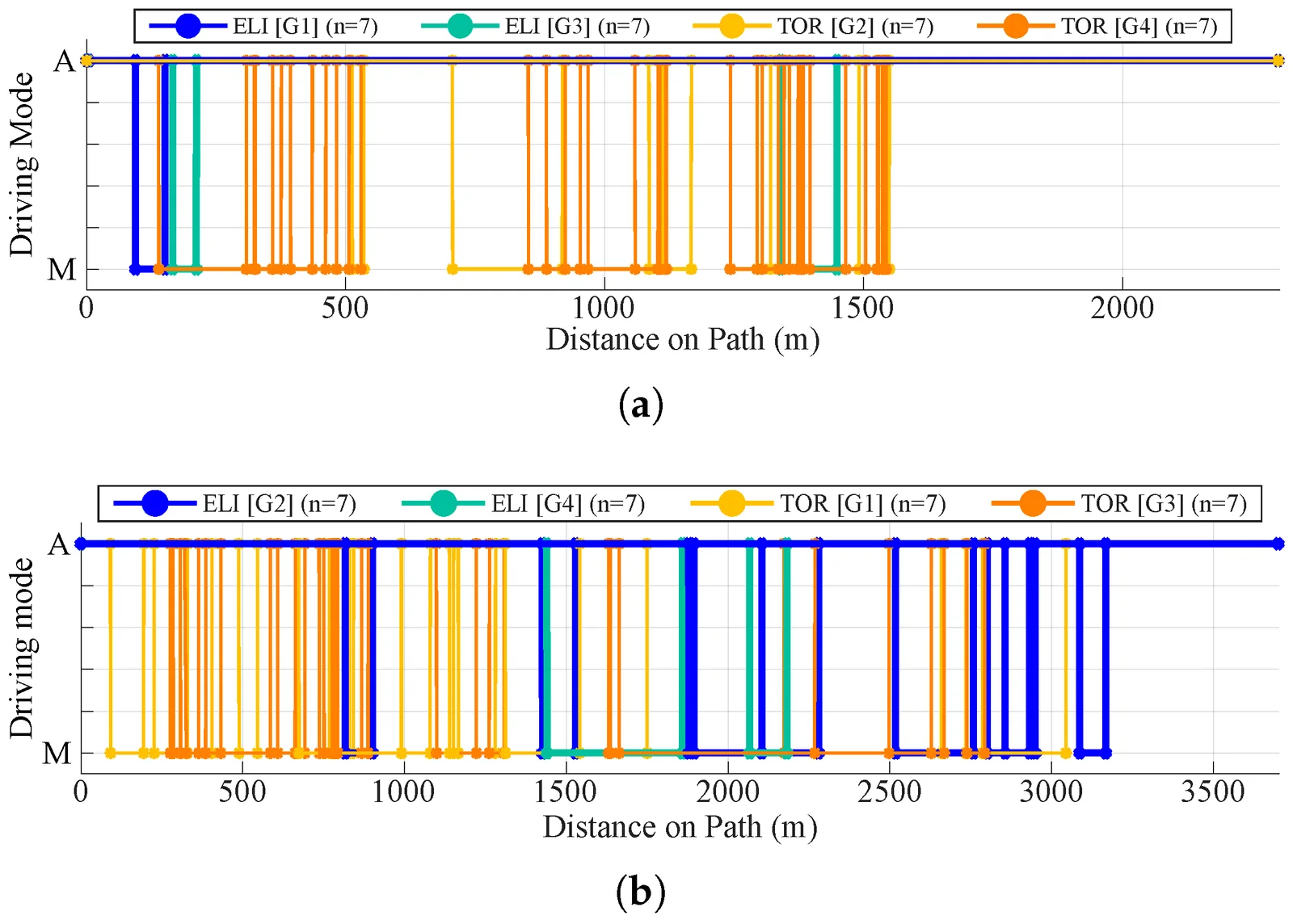
Driving mode—manual (M) or autonomous (A)—during the experiments. (**a**) Urban layout. (**b**) Peri-urban Layout.

Figure 14a complements Table 6 by showing how the driving modes changed throughout the traveled distance in each experiment. Figure 14a presents the behavior observed in the urban layout. Participants using ELI remained in automated mode for nearly the entire route, with only a few transitions to manual driving. In contrast, transitions to manual mode were more frequent among participants assisted by the TOR system.

Figure 14b shows a similar overall pattern in the peri-urban layout, with manual control occurring more frequently under TOR than under ELI. However, between approximately 1500 and 3000 m, participants using ELI transitioned to manual mode more often. This section of the route included several stop-controlled intersections where the ADS came to a complete stop and occasionally remained stationary longer than participants considered necessary, even when no other vehicles were approaching. As a result, some participants took control to continue through the intersection. A traffic light was also located within this section. Although the signal changed from red to green as the vehicle approached, some participants still chose to take control as a precaution, even though no manual intervention was required.

Finally, the latency of the ELI mode is evaluated, since the underlying communication layer must maintain strict temporal performance to operate effectively under these safety-critical fallbacks and dynamically compressed windows. Over the 28 conversational sessions, the system recorded 712 interaction turns. The interaction featured a high volume of verbal engagement, accounting for 498 human utterances (averaging 17.8 per session) compared to 214 ADS utterances (averaging 7.6 per session). Furthermore, the LLM component classified 62.25% of human utterances as in-domain, indicating a high rate of contextually relevant task engagement by the drivers.

Table 7 summarizes the performance of the two LLM-dependent stages of the pipeline. All metrics are computed over the same recorded interactions. The sets are nested: of the 498 classified utterances, 204 were driving commands, of which 163 reached the vehicle and were verifiable. On the other hand, 214 cases correspond to spoken explanations of ADS responses. The domain classifier reached an accuracy of 0.90, with 32 false positives and 14 false negatives over the evaluated set, indicating reliable separation of in-domain driving commands from unrelated speech.

For the generation stages, we report the hallucination rate, defined as the fraction of spoken messages whose asserted frame (action, side, modality, or cause) either contradicts the source ADS message or adds content absent from it. The ADS-response interpretation stage, evaluated per spoken sentence (n=214), showed a hallucination rate of 8.7% (17/214; 95%). The human-command interpretation stage, evaluated by spoken command (n=163), showed a lower rate of 8.6% (14/163; 95% confidence interval: 5.2–13.9). Hallucinations were not uniformly distributed across message types: they concentrated in a small number of response classes, most notably lane_change_unavailable, where the model occasionally fabricated a justification (e.g., stating that a nearby vehicle blocked the maneuver) although the ADS response only indicated that the direction was unavailable, without specifying a cause.

**Figure 15:**
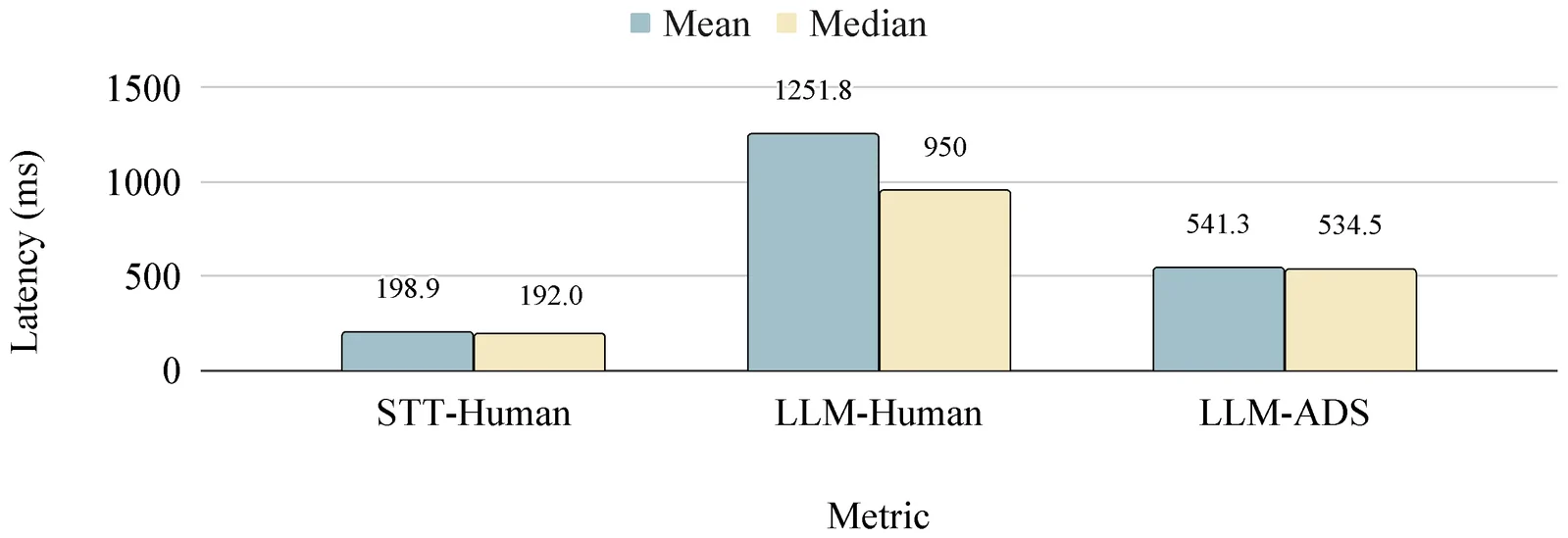
System latency metrics by processing module.

The temporal performance of the ELI pipeline is critical for maintaining the proposed negotiation protocol without jeopardizing the dynamic time budget. The latency analysis, as shown in Figure 15, indicates that the system is reasonably responsive. For processing human utterances, the Whisper-based STT module operated with a mean latency of 198.9 ms (median = 192.0 ms). The subsequent LLM intent interpretation and translation required a mean processing time of 1251.8 ms (median = 950.0 ms). The total end-to-end processing time for human inputs averaged 1451.2 ms (median = 1141.5 ms). The positive skew in LLM processing time (mean > median) indicates that while most inputs are processed in under a second, a subset of more complex queries required additional computational time. On the contrary, the LLM latency for generating ADS utterances was significantly faster, with a mean of 541.3 ms (median = 534.5 ms), ensuring that automated tactical proposals are generated and communicated to the driver with minimal delay.

### 3.2. Physiological Analysis

To evaluate the physiological impact of ELI versus TOR, we analyzed the continuous sensor data across both the urban and peri-urban routes. To account for inherent inter-subject physiological variability, all data were normalized against each driver’s pre-experiment resting baseline. The results are expressed as changes from baseline, where 0 indicates no physiological change relative to the driver’s resting state.

**Figure 16:**
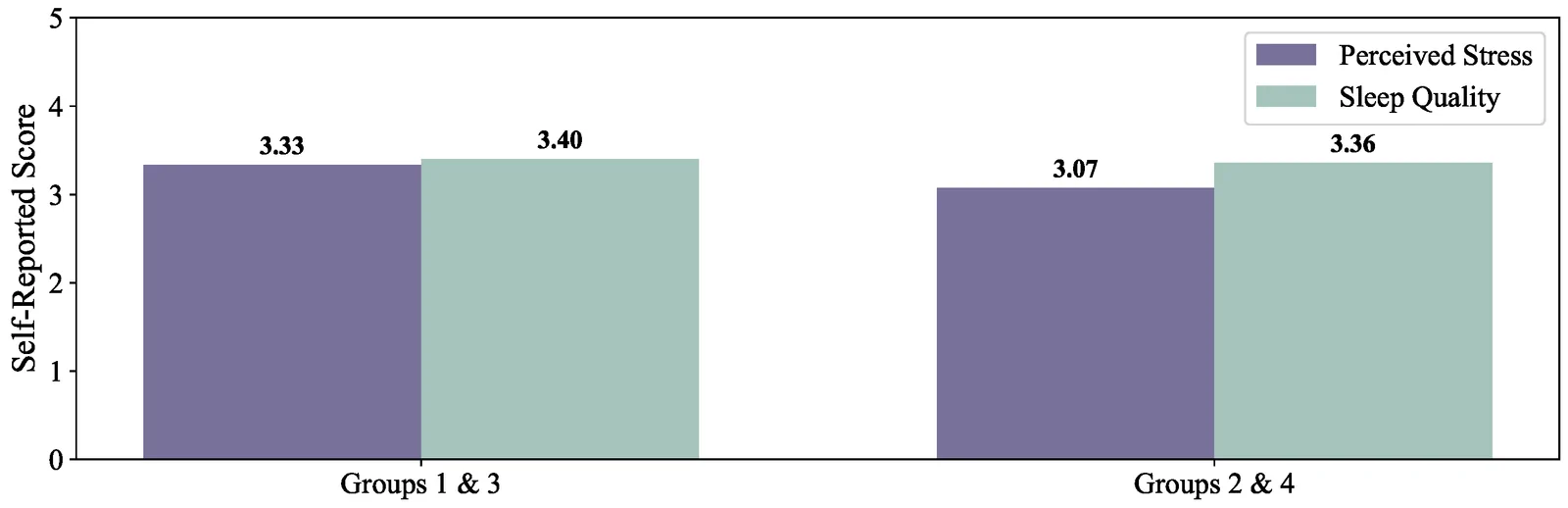
Comparison of stress and sleep of Groups 1 and 3 and Groups 2 and 4.

To improve the robustness of the physiological evaluation, a preliminary analysis of the participants’ self-reported baseline characteristics was conducted using the pre-experiment questionnaire. The analysis of the pre-experiment self-assessments shown in Figure 16 indicated some between-group differences in baseline stress and sleep quality; specifically, Groups 1 and 3 reported higher daily stress levels and lower sleep quality than Groups 2 and 4. Because these baseline differences may introduce an inherent skew in isolated, single-environment time-series comparisons, the subsequent physiological evaluations emphasize aggregated data across both routes.

To understand this necessity, consider the experimental design: in the urban layout, the ELI condition was evaluated by Groups 1 and 3, the cohorts with higher self-reported baseline stress, while the TOR condition was evaluated by Groups 2 and 4. Conversely, in the peri-urban layout, the ELI condition was evaluated by Groups 2 and 4, while the TOR condition was evaluated by Groups 1 and 3. Therefore, examining physiological responses within a single layout may inadvertently compare groups with different baseline profiles. By aggregating the data across both the urban and peri-urban layouts, every participant and baseline profile is represented within both the ELI and TOR datasets. This aggregation helps balance the influence of group differences and provides a more representative comparison of the physiological load across driving frameworks.

**Figure 17:**
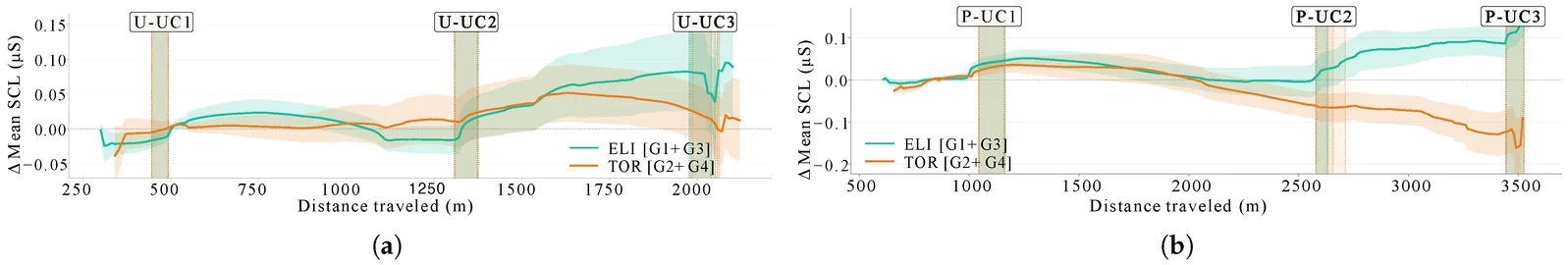
Comparison of skin conductance levels—mean SCL. (**a**) Urban layout. (**b**) Peri-urban layout.

**Figure 18:**
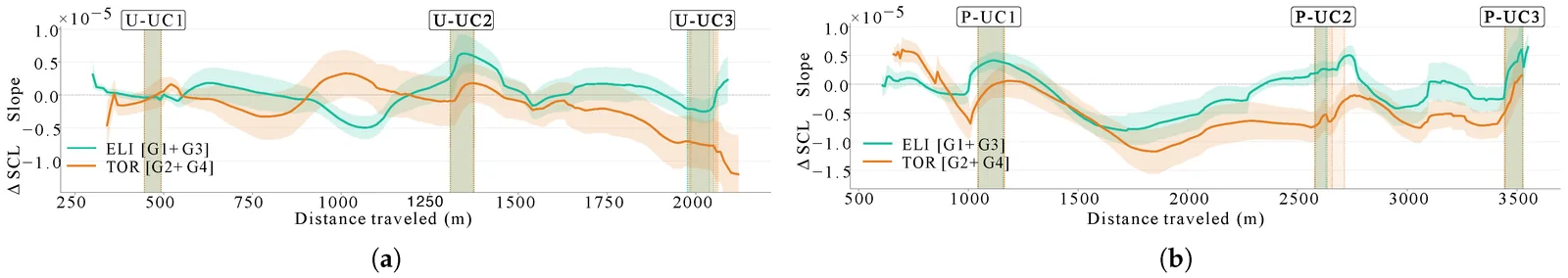
Comparison of skin conductance level (SCL) slopes. (**a**) Urban layout. (**b**) Peri-urban layout.

**Figure 19:**
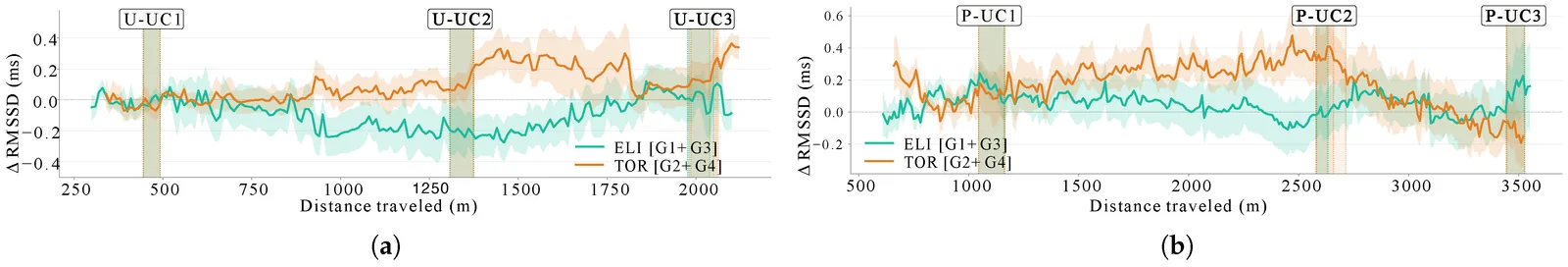
Comparison of RMSSD. (**a**) Urban layout. (**b**) Peri-urban layout.

**Figure 20:**
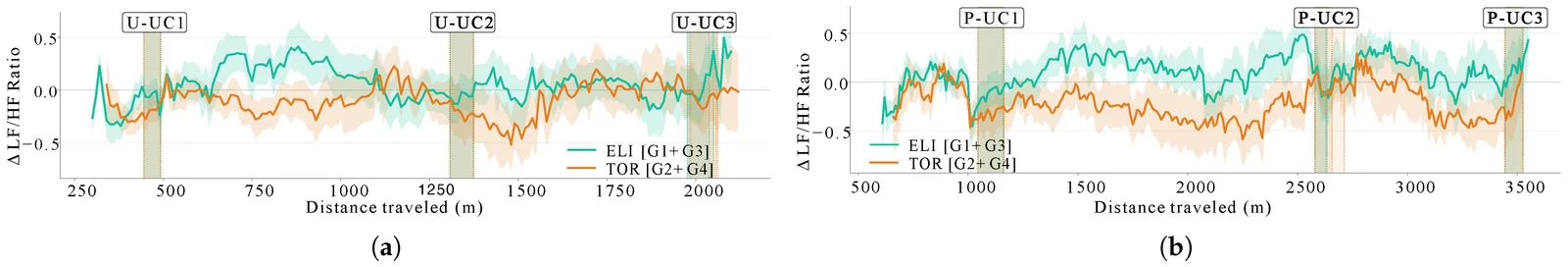
Comparison of HRV LF/HF. (**a**) Urban layout. (**b**) Peri-urban layout.

For temporal analysis across the driving routes, we focused on four physiological metrics: two from EDA and two from HRV, each metric capturing a complementary aspect of the driver’s autonomic state. From the EDA, we analyzed the mean SCL, which reflects the overall level of tonic arousal, with higher values indicating greater sustained activation, and the SCL slope, which captures the direction and rate of that tonic arousal over time, indicating whether the driver is progressively becoming more or less activated. From the HRV, we analyzed the RMSSD, a marker of parasympathetic (vagal) activity that reflects how relaxed and recovered the driver is, and the LF/HF ratio, an index of sympathovagal balance where higher values indicate a shift toward sympathetic dominance and increased physiological activation. Together, these four metrics allow us to characterize both the intensity (mean SCL, see Figure 17), the trend (SCL slope, see Figure 18), the relaxation (RMSSD, see Figure 19), and the overall autonomic balance (LF/HF, see Figure 20) of the driver’s response throughout each scenario.

#### 3.2.1. Electrodermal Activity

Electrodermal activity provides a direct measure of sympathetic arousal and is commonly associated with changes in cognitive load and emotional stress. To capture the temporal evolution of this activation during the driving tasks, we analyzed the SCL slope, which represents the direction and rate of change of the tonic skin conductance level. A positive slope may indicate increasing sympathetic activation, whereas a negative slope may reflect decreasing arousal or recovery [49].

As shown in Figure 17, the ELI mode exhibited higher mean SCL than the TOR mode in both layouts, with the difference increasing along the route. In the peri-urban layout, this divergence became most pronounced in the final third of the route (after P−UC2), where ELI increased while TOR progressively decreased, and the interquartile bands of the two conditions were largely separated. Consistently, the SCL slope (Figure 18) was positive for ELI and negative-to-flat for TOR toward the end of the peri-urban route, indicating a rising tonic arousal under ELI and a declining one under TOR.

Together, the level (mean SCL) and its trend (SCL slope) point in the same direction: under ELI, tonic electrodermal arousal remained above each session’s calm baseline and continued to rise, whereas under TOR, it fell below baseline, with the two conditions diverging most clearly in the demanding final segment of the peri-urban route. This sustained, rising electrodermal arousal under ELI is consistent with a driver who remains actively engaged with the driving task throughout the route, rather than habituating to it.

#### 3.2.2. Heart Rate Variability

To assess parasympathetic (vagal) regulation, we analyzed RMSSD, the root mean square of successive differences between normal-to-normal intervals, which isolates the rapid beat-to-beat variability driven predominantly by vagal activity. In driving contexts, lower RMSSD values may reflect vagal withdrawal associated with increased stress and cognitive demand, whereas higher values may indicate a more relaxed, parasympathetically dominated autonomic state. RMSSD is considered the reference time-domain marker of vagal tone and can be reliably estimated from short analysis windows, supporting its use for tracking within-drive changes in autonomic state [50,51].

On the other hand, to characterize sympathovagal balance, we analyzed the LF/HF ratio, the ratio of low-frequency (0.04–0.15 Hz) to high-frequency (0.15–0.40 Hz) spectral power, conventionally interpreted such that higher values reflect a shift toward sympathetic predominance and increased physiological activation. In driving contexts, elevated LF/HF may accompany heightened arousal and mental demand during demanding events. Because the physiological specificity of this index remains debated, since LF power reflects both sympathetic and parasympathetic influences, we interpret LF/HF cautiously and report it alongside RMSSD as a complementary, more specific vagal marker [50,52].

As shown in Figure 19, the ELI mode exhibited lower RMSSD values (green) than the TOR mode (orange) across most of the route in both the urban and peri-urban scenarios, indicating reduced beat-to-beat variability under ELI. Consistently, the LF/HF ratio (Figure 20) was higher for ELI than for TOR, reflecting a shift toward sympathetic predominance. The confidence bands of both conditions overlapped along most of the route, with a more pronounced divergence toward the end of the peri-urban route around the critical use cases (UC2–UC3).

#### 3.2.3. Overall Aggregated Physiological Load

**Figure 21:**
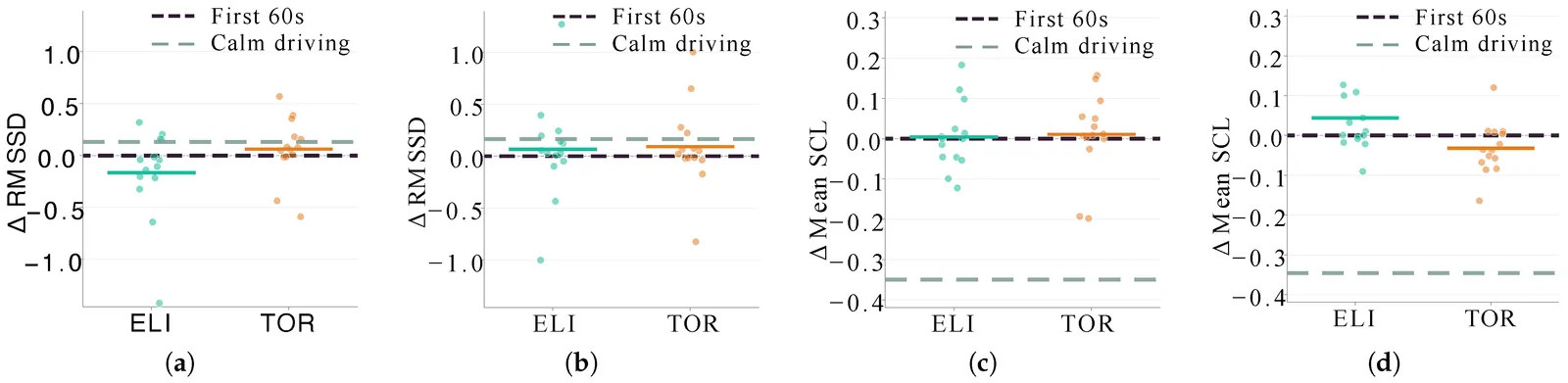
ELI and TOR comparison over physiological data (**a**) RMSSD urban. (**b**) RMSSD Peri-urban. (**c**) Mean SCL urban. (**d**) SCL peri-urban. Green dots correspond to ELI subjects and orange to TOR subjects. The solid lines represent the median value of each set of subjects.

To summarize these dynamics at the participant level, each session was aggregated into a single value per subject: the median of the normalized feature over the whole drive. Figure 21 shows these per-subject values for ELI and TOR, where each point is one participant, the solid horizontal line marks the median of each condition, the dashed line indicates each session’s first 60s baseline (the zero reference), and the grey dashed line marks the calm-driving reference. Within this standardized framework, lower RMSSD reflects reduced vagal tone (greater autonomic activation), while higher mean SCL reflects greater electrodermal arousal. RMSSD was lower under ELI than under TOR in both layouts. Mean SCL separated the conditions most clearly in the peri-urban route, where the ELI median lay above and the TOR median below the session baseline, whereas in the urban layout the electrodermal difference was small. In both conditions, the electrodermal values remained above the calm-driving reference.

We can observe from the charts that ELI, in general, required a higher cognitive load from the subjects, as the deflection of the RMSSD from the baseline was lower than that of users using TOR (see Figure 21a,c). On the other hand, it can also be observed that ELI maintained the subjects with a higher level of sympathetic arousal, which could indicate that they did not disengage from the driving loop.

### 3.3. Driver Perception Analysis

To assess the impact of the conversational interface on user perception, participants evaluated their trust in both the ELI and the baseline TOR across four key dimensions: understanding the system state, predictability of the system’s reactions, logical interpretation of traffic situations, and ease of tracking indications and operational limits. This evaluation was performed through the post-experiment survey, as detailed in Section 2.4.5.

**Figure 22:**
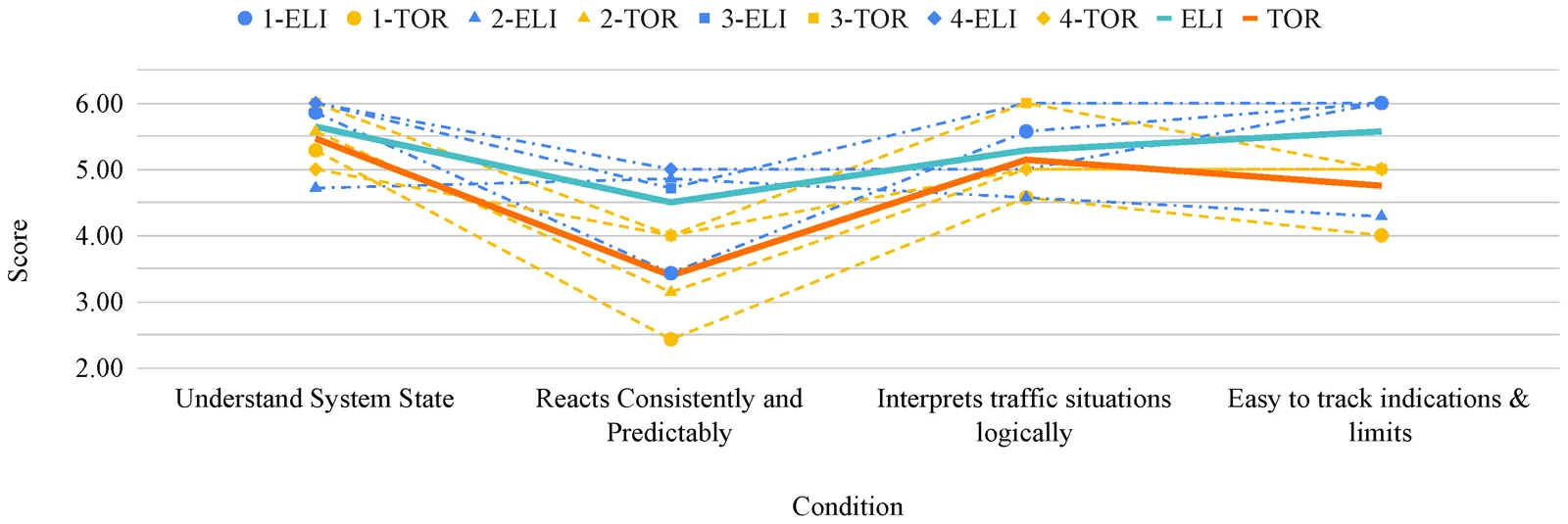
Comparison of subjective driver evaluation scores between the ELI and TOR conditions.

As illustrated in Figure 22, the ELI condition generally yielded higher subjective ratings across experimental groups than the TOR baseline. The natural-language feedback provided by ELI notably enhanced participants’ ability to track system limits and understand the vehicle’s driving logic. The aggregate trend demonstrates that the transparent, bidirectional communication facilitated by ELI improves the human driver’s overall confidence, calibration of predictability, and situational awareness during highly automated driving tasks.

To further examine the questionnaire results presented in Figure 22, the Wilcoxon signed-rank test and the paired-samples Student’s *t*-test were applied to the ELI and TOR ratings from the same participants. Wilcoxon evaluates whether the direction and ranked magnitude of the paired differences favor one mode without assuming a normal distribution, while the paired *t*-test evaluates whether the mean numerical difference between ELI and TOR differs from zero. Together, these tests provide nonparametric and parametric comparisons of the observed responses.

Table 8 presents the Wilcoxon signed-rank results. ELI and TOR are summarized by the median and the interquartile range [Q1–Q3], where Q1 is the value below which 25% of the responses fall and Q3 is the value below which 75% fall; therefore, [Q1–Q3] contains the middle 50% of the responses. The paired difference is defined as $\Delta_{ET}=\mathrm{ELI}-\mathrm{TOR}$, so positive values favor ELI. $W^{+}$ is the sum of the ranks for positive differences, the *p*-value evaluates whether the paired differences show a systematic displacement from zero, and $r_{\mathrm{rb}}$ gives the direction and magnitude of the rank-biserial effect, with positive values favoring ELI.

As shown in Table 8, all four rank-biserial effects were positive, showing a tendency toward ELI. The largest difference occurred for consistency and predictability. Although both modes had a median of 5, the middle 50% of the ELI responses ranged from 4 to 6, compared with 2.75 to 6 for TOR. This item also showed $\Delta_{ET}=0.5$, the highest positive-rank sum ($W^{+}=178.5$), statistical significance when considered individually (p=0.026), and the largest effect ($r_{\mathrm{rb}}=0.545$). The other items were not significant, although their positive effects also favored ELI ($r_{\mathrm{rb}}=0.233$–0.395).

Table 9 presents the paired-samples Student’s *t*-test results. ELI and TOR are summarized by the mean and standard deviation. Here, $\Delta_{ET}$ is the mean within-participant difference, with positive values favoring ELI. The *t* statistic compares this mean difference with its variability across participants, while the *p*-value evaluates whether the mean difference is different from zero. Cohen’s $d_{z}$ represents the paired effect size, with positive and larger values indicating a stronger effect toward ELI.

Consistent with the Wilcoxon results, ELI showed higher mean ratings in all four items, as indicated by the positive $\Delta_{ET}$ values. The largest difference was again found for consistency and predictability, with means of 4.93 for ELI and 4.11 for TOR ($\Delta_{ET}=0.82$). This item had the largest *t* statistic, t(27)=2.43, was significant when evaluated individually (p=0.022), and showed the largest paired effect ($d_{z}=0.460$). The other mean differences also favored ELI ($\Delta_{ET}=0.21$–0.61) but were not significant.

To evaluate whether presentation order influenced the participants’ evaluations, a Mann–Whitney *U* analysis was conducted. Participants were divided into two independent sequences: TOR-first (Groups 1 and 2, n=14) and ELI-first (Groups 3 and 4, n=14). For each participant and item, $\Delta_{ET}=\mathrm{ELI}-\mathrm{TOR}$ was calculated and compared between the two sequences to determine whether the ELI–TOR differences varied with presentation order. The results are shown in Table 10.

As shown in Table 10, no significant differences were found between the TOR-first and ELI-first sequences for any questionnaire item (p>0.05). The mean $\Delta_{ET}$ values were generally positive in both sequences, showing that ELI tended to receive higher ratings regardless of order. Understanding the system state was the only item with opposite mean directions between sequences, but this difference was not significant (p=0.195). Thus, no detectable evidence of presentation order or carryover effects was observed.

### 3.4. Driving Performance

ELI is a human–machine collaboration interface designed to keep the human involved in the driving loop even when the vehicle is driven in autonomous mode. By keeping the human in the loop, they can participate in decision-making, improving safety and driving while making them feel more comfortable. This section analyzes the driving performance of different driving groups across different driving modes and layouts.

To analyze driving performance, we used the sensor data from the simulations and conducted an extensive comparison of several key performance indicators (KPIs), including comfort metrics (longitudinal and lateral accelerations and jerks) and safety metrics (risk of close encounters, number of collisions, and out-of-lane events). Table 11 presents the detailed formulas for computing each KPI.

The comfort metrics are computed as the mean of the absolute values of the accelerations and jerks along the travel path. In the case of the safety metrics, three values were considered: the number of collisions (with other vehicles or with static objects), the number of out-of-lane events, and the risk to collision, which is computed from the risk from time to close encounter (RTTCE) metric proposed in [53], obtained during the travel considering the position of the ego-vehicle with respect to the obstacles present on the scene. The considered metric uses the sum of the maximum risk value over the trajectory and the average risk value.

**Figure 23:**
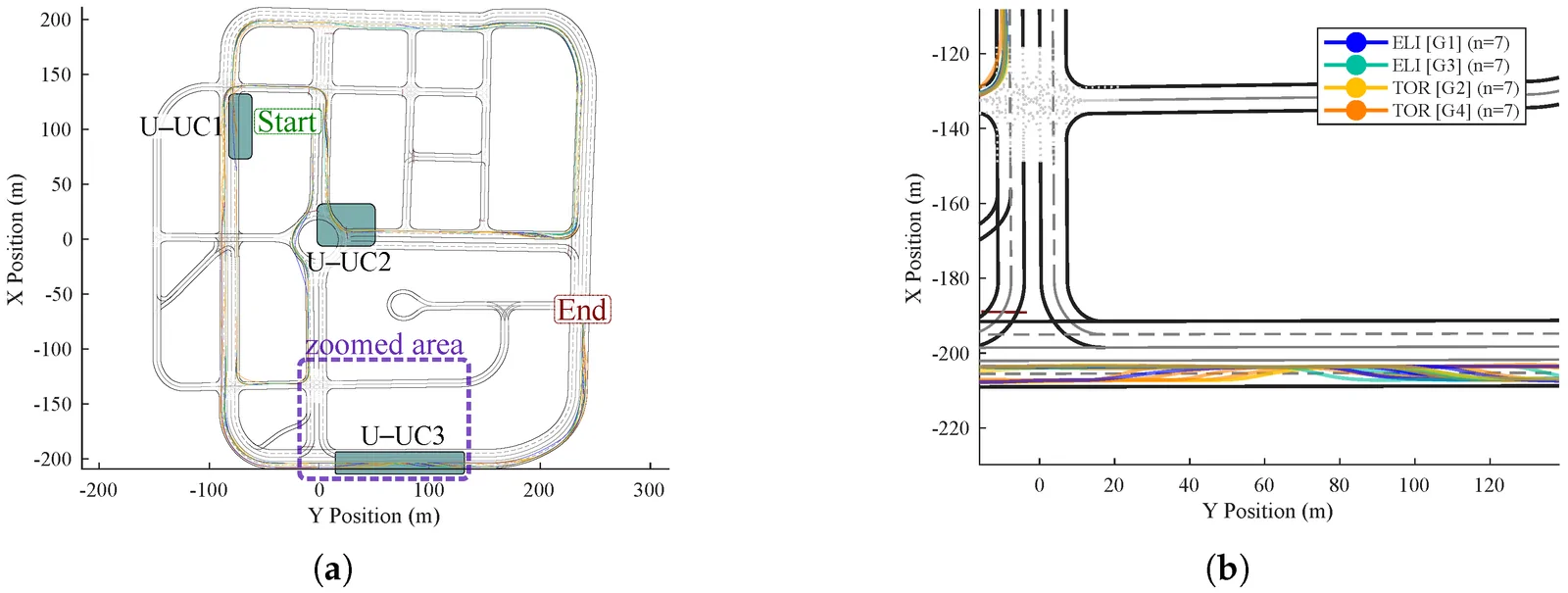
Paths of the experiments on the urban layout. (**a**) Complete travel. (**b**) driving paths during the use case U−UC3.

Figure 23 shows the travels performed for the urban layout. Blue/cyan lines represent the groups that performed the experiment in ELI mode [G1 and G3], while orange/yellow lines represent the groups that performed the experiment in TOR mode [G2 and G4].

Figure 23a shows the complete layout, including the start and end positions and the locations of the three use cases depicted in Figure 7. On the other hand, Figure 23b shows a zoomed-in view of use case U−UC3 (detailed in Figure 8). The figure shows that users in ELI mode tend to make the right-lane change earlier than TOR users.

**Figure 24:**
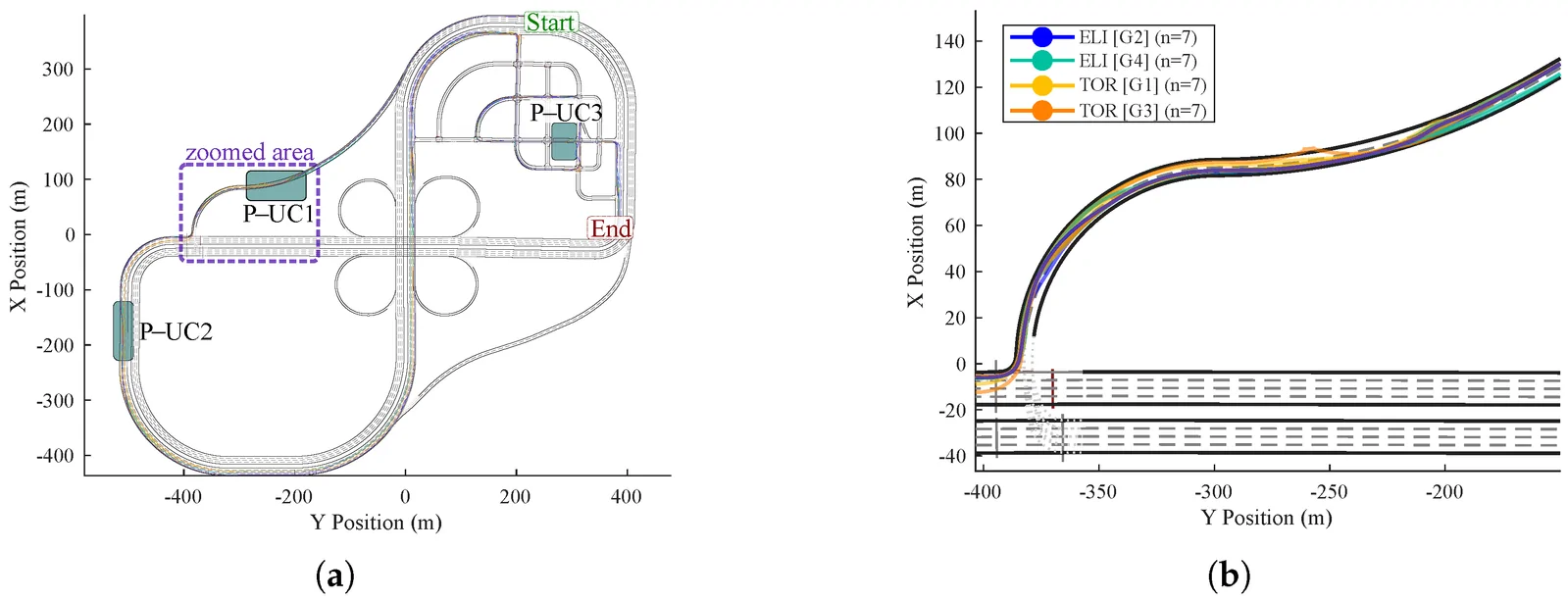
Paths of the experiments on the peri-urban layout. (**a**) Complete travel. (**b**) Paths during the first risk situation.

Figure 24 shows the travels performed for the peri-urban layout. It can be observed that the first part of the layout consists of a highway, while the latter part is developed in a suburban area, where the use of P−UC3 takes place. For this layout, the groups for ELI mode were [G2 and G4], while the groups that performed the experiment in TOR mode were [G1 and G3].

Figure 24a shows the complete layout and the locations of the three use cases shown in Figure 9. In this case, Figure 24b shows a zoomed-in visualization of use case P−UC1 (depicted in detail in Figure 10). The figure shows that, once more, ELI users make the lane change earlier than TOR users to avoid the obstacle.

**Figure 25:**
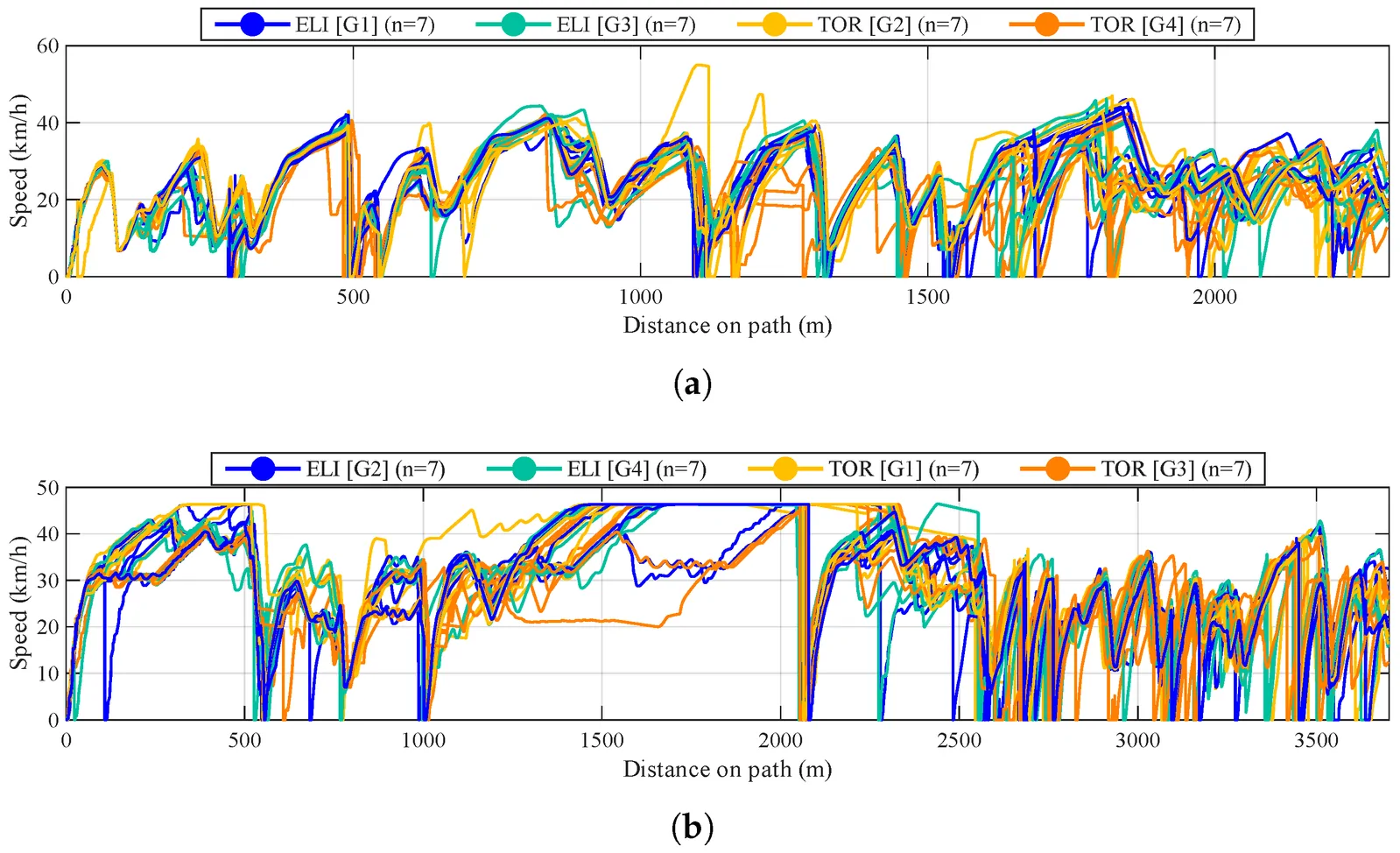
Speed profiles of the driving experiments. (**a**) Urban layout. (**b**) Peri-urban Layout.

As the comfort metrics depend directly on the ego-vehicle speed along the route, Figure 25 shows the speed profile for each experiment performed in this study. It is worth mentioning that almost all profiles exhibit similar behavior throughout the trip. This is because the system is in autonomous mode most of the time. Nevertheless, the figure shows that in both layouts, outliers indicate different driving behavior, achieving higher or lower speeds than in the other experiments. In the urban layout (Figure 25a), the yellow line reaches a higher speed at approximately 1100 m. In the case of the peri-urban layout, there is another yellow line that reaches higher speeds around 1000 m, and one orange line that shows lower speeds than the rest at approximately 1300 m. In the three mentioned cases, the vehicle was being driven in manual mode.

After extracting telemetry and sensor data from the experiment logs, we computed the KPIs using the equations detailed in Table 11 to facilitate a comparative analysis of the driving modes. To objectively visualize the performance of the system, we used a 7-axis radar chart normalized to a “smaller-is-better” scale.

**Figure 26:**
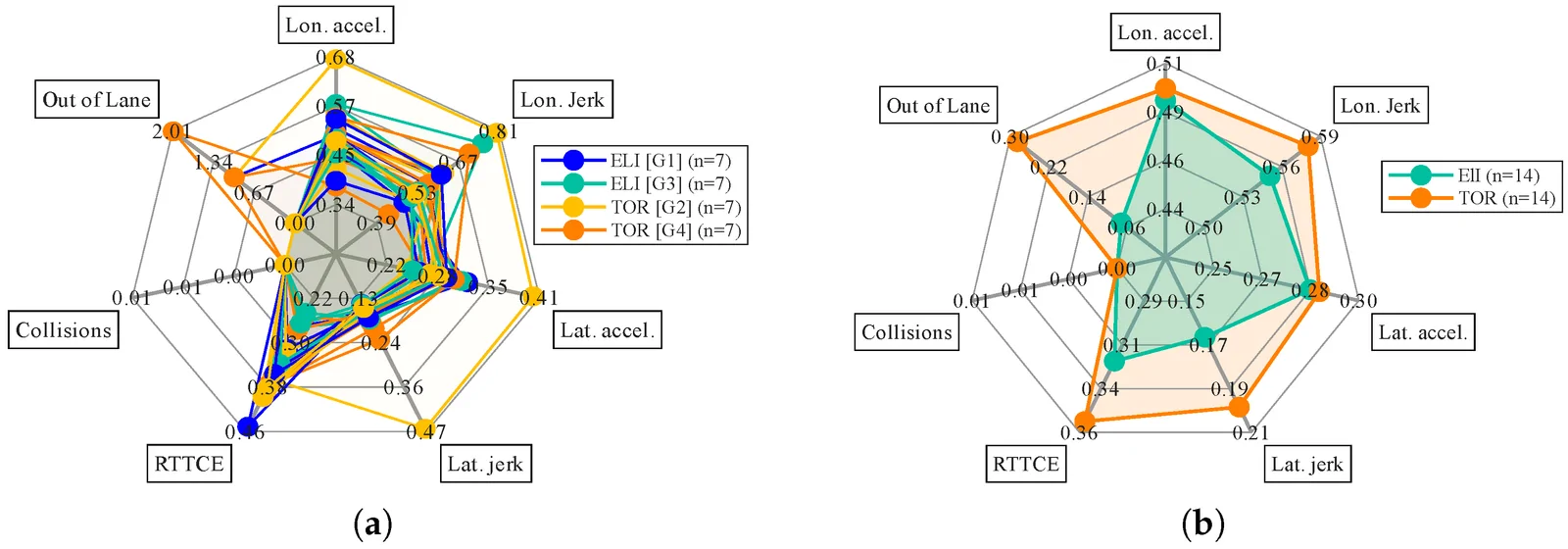
KPIs of the experiments on the urban layout. (**a**) KPIs for all experiments. (**b**) Mean KPI values per driving group.

Figure 26 shows the experimental analysis for the urban layout. We plotted the individual KPIs for each subject according to their respective experimental group (G1, G2, G3, and G4) (see Figure 26a) and calculated the aggregate mean values for both the ELI and TOR driving modes (see Figure 26b).

The radar charts reveal several notable trends. Overall, the ELI mode yielded slight improvements over the TOR baseline across all measured KPIs. The most significant discrepancy was observed in the “out-of-lane” safety metric, where the TOR mode recorded four times as many lane-departure events as the ELI mode. Furthermore, the average RTTCE was lower under the ELI condition, indicating that the ego-vehicle maintained a greater safety margin from obstacles throughout the route. Notably, no collisions were recorded across any of the experimental runs in either mode. In terms of physical comfort, longitudinal and lateral acceleration and jerk metrics were comparable between the two systems, indicating that the ELI mode achieves a smooth dynamic driving profile similar to that of manual driving.

**Figure 27:**
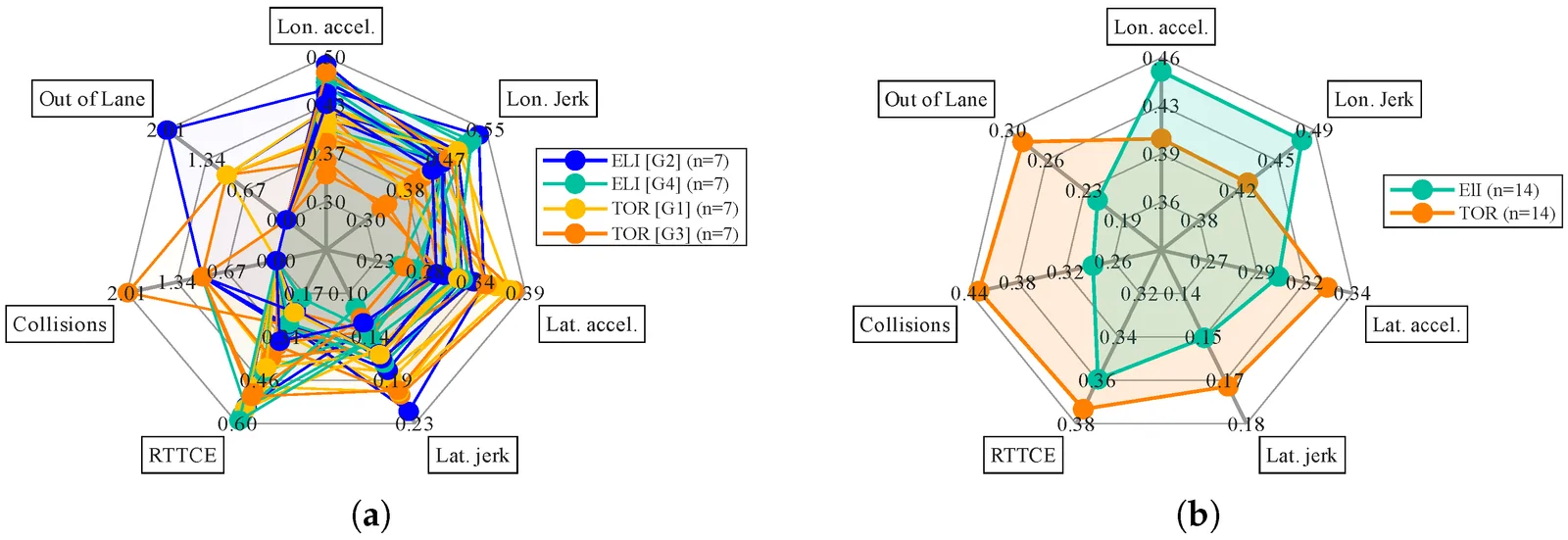
KPIs of the experiments on the peri-urban layout. (**a**) KPIs for all experiments. (**b**) Mean KPI values per driving group.

Figure 27 illustrates the performance radar charts for the peri-urban layout. Consistent with previous findings, the ELI mode demonstrated superior safety performance across all metrics compared to the TOR baseline. While collisions with static obstacles occurred under both conditions (six for TOR and four for ELI), a critical distinction is that all collisions recorded during the ELI trials occurred while the vehicle was operating in manual mode. This indicates that the ELI system itself successfully avoided collisions, and the recorded accidents likely stem from human error following a voluntary handover. More specifically, two collisions occurred after participants assumed manual control in the middle of a turn, whereas the other two occurred when participants took control while entering the highway and subsequently collided with a roadside obstacle. In the latter cases, the collisions were associated with difficulties in judging the vehicle’s lateral position and the available surrounding space immediately after the transition to manual driving. These events therefore suggest that the observed collisions were more closely related to the driver’s control and spatial awareness after the handover than to the autonomous behavior of ELI, potentially due to the complexity of the peri-urban environment or transition-induced workload. Furthermore, the ELI mode yielded a lower RTTCE. This improvement is likely attributable to the system executing earlier, more proactive lane changes in these use cases. Consequently, ELI registered fewer lane-departure events than TOR mode. In terms of physical comfort, the metrics remained largely comparable; however, the TOR mode exhibited slightly better longitudinal stability. This reflects a minor, but expected, trade-off, as ELI’s cautious, proactive maneuvering to prioritize collision avoidance inherently introduces slight variations in longitudinal vehicle dynamics.

### 3.5. Thematic Analysis

In order to holistically evaluate users’ perceptions of the ELI and TOR systems, a thematic analysis of their qualitative responses was conducted. This allowed us to better understand the quantitative metrics above. The analysis process was performed on responses to the following two prompts:

- Describe how you would use and trust the ADS mode in situations where you are distracted and need to take control.
- Describe how you would use and trust the ELI mode in situations where you are distracted and need to take control.

It began with translating all Spanish responses into English. Initial codes were generated by identifying recurring concepts across the text, such as “feedback,” “alertness,” “delay,” “hands-free,” or “understanding.” These initial codes were then systematically applied across the dataset and grouped into distinct themes using pattern recognition. The themes were identified to align coherently with the study’s predefined hypotheses and validated against participant data to ensure accurate representation.

Theme 1: Conversational Engagement and Active Monitoring (Link: H1): This theme captures how the ELI system’s conversational nature maintained driver engagement within the operational loop. Voice interactions provided an accessible way for users to remain active without requiring physical intervention, except in critical situations. Eight participants indicated that the ELI conversational interface facilitated sustained attention and enabled rapid responses. Some quotes that support this theme are as follows:

- “I like this mode because it is easier and faster to give a voice instruction in an emergency, whether to stop, continue, turn, etc., and the system reacts quickly.” (Subject 24)
- “I would trust it, but I would be paying attention all the time to be able to speak and react correctly when taking control.” (Subject 19)

Theme 2: System Transparency and Shared Awareness (Link: H2): Participants noted that the ELI interface improved their comprehension of the system’s intended actions, thereby increasing their situational awareness. Unlike the TOR system, where users cited a lack of feedback as a primary reason for distrust, ELI’s conversational checks allowed drivers to verify the system’s logical interpretation of traffic scenarios. Five participants explicitly connected the ELI system’s verbal feedback to a better understanding of its decisions. Some quotes that support this theme are as follows:

- “In this mode, autonomous control is more reliable, because as it is a conversation, you feel more sure of the decisions that are made.” (Subject 26)
- “I feel more comfortable with this system because it calls my attention when it speaks, so it would be easier for me to take control with the feedback it is giving me.” (Subject 7)
- Contrast with TOR: “If this mode asks me to take control, I wouldn’t trust it much, because I don’t know about the decisions it can make.” (Subject 26)

Theme 3: Perceived Trust and Supervised Autonomy (Link: H3): The qualitative responses demonstrate that ELI fosters trust while mitigating the risk of over-reliance, in line with other aspects that impact trust, e.g., safety and comfort results [54,55] (See Section 3.4), Ten participants reported positive confidence in the ELI mode while maintaining a clear awareness of its limitations, such as processing delays or domain-specific limitations (e.g., highways versus urban areas). Drivers expressed a willingness to rely on the system while consistently acknowledging the necessity for continuous supervision. Some quotes that support this theme are as follows:

- “I would use it with confidence but still pay attention to the road.” (Subject 21)
- “The ELI needs approval before actually making decisions… the fact that it checks is very helpful in case of a bad situation.” (Subject 17)
- “It works well, it understands your voice commands well, but there was a lot of delay between its questions and the situations, so on some occasions I felt insecure.” (Subject 3)

Theme 4: Physical Comfort and Perceived Safety (Link: H4): Participants perceived the ELI mode as a tool that provides physical comfort and practical safety support, particularly during fatigue or when manual control is obstructed. Five participants reported that the voice-activated system increased physical ease and safety compared to the traditional nonverbal baseline.

- “I would use it if I were tired or my hands were occupied.” (Subject 28)
- “I will trust more in this system, even at stop signs and if I’m sleepy.” (Subject 16)
- “I felt very comfortable, and it responded to the commands; I would feel comfortable using it.” (Subject 18)

### 3.6. Limitations

Several limitations must be acknowledged when interpreting the findings of this study. First, while the sample size of 28 participants is standard for simulator-based human-in-the-loop studies, it inherently limits the statistical power of the analysis. This is particularly relevant when evaluating highly variable subjective responses and physiological measures, which can vary significantly among individuals. Aside from the sample size, our sample demographics may not be fully representative of the general population. Our results can serve as a valuable baseline for future evaluation of possible design choices for specific user groups, including novices and users with disabilities.

Second, the reliance on the CARLA driving simulator restricts the ecological validity of the findings. Although this virtual environment enabled safe, reproducible testing of complex scenarios, it cannot fully replicate the sensory feedback, physical risks, and psychological pressures inherent in real-world driving. Consequently, participants’ perceived risk and subsequent interactions with the automated driving system may differ from their behavior in a physical vehicle.

Third, the duration and structural scope of the experiment may have influenced the interaction dynamics. While the designed routes successfully captured acute interventions, they represent a relatively brief exposure to the system. Trust calibration and human–AI teaming behaviors are dynamic processes; therefore, longer-term longitudinal studies across a wider range of operational domains are necessary to understand how a driver’s reliance on and engagement with the ELI system evolve over time.

Finally, technical constraints within the voice interaction pipeline introduced practical limitations. Variations in human speech, such as inadequate microphone pickup or unclear enunciation, occasionally led to speech recognition errors or misinterpretations by the LLM. These situations occasionally disrupted the natural flow of negotiation and delayed system responses, which, as noted in the subjective feedback, can temporarily degrade user confidence and affect the timing of safety-critical interventions.

## 4. Discussion

The transition from conditional automation to collaborative autonomy requires a paradigm shift in how vehicles and human operators interact. The primary objective of this study was to investigate and establish novel principles for effective human–AI teaming in safety-critical, dynamic environments, using highly automated vehicles as a primary application domain. By evaluating the ELI system against a traditional TOR baseline, this research demonstrates that a bidirectional, LLM-powered communication layer fundamentally alters the driver’s role. The multidimensional findings (spanning negotiation latencies, physiological responses, subjective trust, and vehicular driving performance) provide comprehensive support for our four working hypotheses. Instead of acting as a passive fallback agent, the driver becomes an active teammate in a dynamic, joint decision-making loop.

Addressing the first hypothesis (H1), the data confirm that a conversational interface keeps drivers actively engaged without inducing cognitive overload. During the simulator experiments, participants interacted frequently, averaging 18 utterances per experiment, and over 62% of their utterances were accurately classified as in-domain task engagement. When collaborative decisions were required, the times were reasonably bounded, with a median of 1.1 s for the full conversation cycle, fitting within the system’s dynamic time budgets. The physiological data were consistent with this behavioral engagement. Under ELI, SCL remained mildly above each session’s baseline, indicating a sustained but moderate tonic arousal. Under the TOR baseline, tonic arousal instead declined below baseline during automated segments, consistent with a more disengaged state. While a reduced tonic arousal might seem benign, at this level of automation it may reflect a miscalibration of trust (over-reliance), since the driver is still expected to remain ready to respond to unexpected events. Conversely, the arousal increase under ELI remained mild and sustained rather than reaching overload levels, suggesting that the conversational interface preserved driver engagement without imposing an excessive physiological cost.

Similar to other human–automation research studies [54,55], this sustained engagement was associated with higher perceived situational awareness (H2) and better-calibrated trust (H3). Participants consistently rated ELI higher for making the system’s operational state understandable and for logically interpreting complex traffic. This pattern was also reflected in both statistical analyses, which showed positive ELI–TOR differences and effect sizes across all four questionnaire items, with the strongest effect observed for consistency and predictability. These results provide additional support for H2 and H3 by showing that the favorable perception of ELI was consistent across the evaluated aspects. By offering natural-language feedback, ELI allowed drivers to verify the vehicle’s reasoning before a maneuver was executed, bridging the transparency gap that normally occurs on traditional TOR systems. Importantly, this confidence did not turn into dangerous complacency or overtrust. Drivers recognized the system’s limits, acknowledged the occasional processing delays, and kept a “supervised autonomy” mindset. The HRV results pointed in the same direction as the electrodermal ones, which is notable because the two measures capture autonomic activation in opposite ways. On both routes, ELI showed lower RMSSD than TOR, a sign of reduced vagal (parasympathetic) tone, and at the same time a higher LF/HF ratio, which suggests a shift toward sympathetic activation. Because a parasympathetic and a sympathetic indicator agree, we can be more confident that the difference reflects a real, if small, rise in activation under the conversational mode. This pattern held on both layouts, and since each participant drove each route only once, it cannot be explained by drivers becoming familiar with a specific route; it is more plausibly linked to the interaction mode itself. In all cases, the increase in activation was small, in line with our reading of ELI as keeping drivers engaged at a modest physiological cost.

Ultimately, active teaming made the driving environment safer, strongly supporting the fourth hypothesis (H4). Telemetry and sensor logs revealed that the ELI mode outperformed the TOR baseline across critical safety indicators. The conversational system produced four times fewer out-of-lane events and maintained a lower average risk from time to close encounter, indicating greater safety margins around obstacles. A particularly interesting finding emerged in the peri-urban layout: every collision recorded in the ELI group occurred exclusively while the vehicle was operating in manual mode. The autonomous framework itself successfully avoided collisions, although these events show that safety can still be affected after the transition to manual control. However, across the evaluated safety and driving metrics, ELI showed better overall performance, which supports H4 despite the limitations observed during manual transitions. While the TOR mode exhibited slightly better longitudinal comfort, this is a minor and expected trade-off. ELI prioritizes proactive safety, executing early lane changes that naturally introduce slight dynamic variations to maximize physical security. Nevertheless, it is necessary to adjust the system so that users don’t feel the need to take manual control in ELI mode.

When placed in a broader context, these findings challenge the industry’s reliance on deterministic, alert-based handoffs. Transferring control to a disengaged driver during a complex scenario is inherently risky. By successfully constraining LLMs to act as real-time semantic interpreters within a bounded control vocabulary, we bridge the gap between natural-language processing and tactical vehicle control. This transforms the concept of the operational design domain. Instead of relying solely on geographic limits, we can extend the system’s capabilities by using human cognition to resolve perceptual ambiguities through fluid dialogue.

Moving forward, future research must expand the findings of this study through larger-scale studies to establish a stronger statistical foundation for evaluating the complex physiological states associated with human–AI teaming. To complement biometric data, we will incorporate standardized post-experiment analyses, such as the NASA-TLX, to explicitly quantify cognitive workload. Furthermore, subsequent experimental designs will feature significantly longer, continuous driving sessions to evaluate how a driver’s trust and reliance evolve over an extended timescale. On the technical front, improving ELI’s end-to-end response times remains a primary objective to ensure maximum efficiency during split-second tactical scenarios in real SAE level 3 applications (see Figure 15), the latency was dominated by the language-understanding stage (LLM-Human, 1252 ms). One strategy we already applied at this step was to minimize the output length. Another popular option is streaming; however, in a driving context, it is inapplicable, since the ADS requires a complete, validated command before execution. Latency reduction must therefore come from the model and inference side. Prior work offers several avenues: speculative decoding, which drafts tokens with a small model that the target model verifies [56]; weight quantization and pruning to reduce the memory footprint [57]; and knowledge distillation into smaller models, which recent work shows can preserve most of the task performance at a fraction of the size [57]. We also plan to expand the system’s collaborative capabilities, allowing drivers to verbally adjust core automated parameters (such as traveling speed, driving behavior profiles, and preferred safety distances) in real time. Finally, deploying this framework in real-world, highly automated vehicles, potentially integrated with multimodal inputs such as gaze or gesture tracking, will be essential for validating the interface against authentic physical risks and varied acoustic environments.

## 5. Conclusions

The results support the four hypotheses proposed in this study. ELI maintained drivers involved during automated operation, increased their awareness of the vehicle’s actions, surrounding traffic, and system limitations, and promoted trust while preserving a supervisory role. It also improved overall driving performance across the evaluated safety metrics by maintaining safer margins and reducing critical driving events, although the ADS showed slightly better longitudinal comfort. Taken together, these findings indicate that conversational interaction can support a more active, informed, and safety-oriented collaboration between drivers and automated vehicles under the conditions evaluated in this study.

## Figures and Tables

**Table 1:** Parameters for the time budget computation of the negotiation protocol.

Parameter	Value
$t_{\mathrm{crit}}$	2 s
$t_{\mathrm{safe}}$	7 s
$S_{\mathrm{thd}}$	0.9

**Table 2:** Categorical demographic characteristics of the participants.

Variable	Category	N	Percent
Gender	Female	13	46.4
	Male	15	53.6
Experimental Group	Group 1	7	25.0
	Group 2	7	25.0
	Group 3	7	25.0
	Group 4	7	25.0
ADAS/AV Experience	None	16	57.1
	Theoretical	10	35.7
	Practical	2	7.1

**Table 3:** Numerical demographic and self-reported characteristics of the participants.

Variable	Min	Max	Mean	Median	SD
Age (years)	18	46	26.64	24.5	6.75
Driving Experience (years)	1	26	6.54	5	5.65
Sleep Duration (hours)	4	8	6.43	7	0.84
Sleep Quality	1	5	3.43	4	1.03
Stress Level	1	5	3.21	3	1.03

**Table 4:** Assignment of participants across assistance modes and driving layouts.

Group	Experiment 1	Experiment 2	Participants
G1	Peri-urban + TOR	Urban + ELI	7
G2	Urban + TOR	Peri-urban + ELI	7
G3	Urban + ELI	Peri-urban + TOR	7
G4	Peri-urban + ELI	Urban + TOR	7

**Table 5:** Summary of multimodal features used in the attention estimation system.

Signal Type	Feature Category	Extracted Features
ECG	HRV frequency domain	LF/HF ratio, RMSSD
EDA	Tonic SCL	Mean SCL, SCL slope

**Table 6:** Manual mode occurrences for each assistance mode under the driving layouts.

Layout	ELI		TOR	
	Manual Mode Events	Number of Users Manual Mode	Manual Mode Events	Number of Users Manual Mode
Urban	3	2	28	8
Peri-urban	16	7	34	13

**Table 7:** Evaluation of the LLM pipeline across stages, over the same interaction data.

Stage	Metric	Value	*n*
Domain classification	Accuracy	0.91	498
	False positives	32	498
	False negatives	14	498
Maneuver interpretation	Maneuver error	0.5% (1/204)	204
Human-command interpretation	Hallucination rate	8.6%	163
ADS-response interpretation	Hallucination rate	8.7%	214

**Table 8:** Wilcoxon signed-rank comparison between ELI and TOR.

Questionnaire Item	ELI Med [Q1–Q3]	TOR Med [Q1–Q3]	$\Delta_{\mathrm{ET}}$ Median	$W^{+}$	*p*	$r_{\mathrm{rb}}$
Understanding system state	6.0 [5.0–7.0]	6.0 [5.0–6.0]	0.0	74.0	0.453	0.233
Consistency and predictability	5.0 [4.0–6.0]	5.0 [2.75–6.0]	0.5	178.5	0.026	0.545
Logical interpretation	5.5 [5.0–7.0]	5.0 [4.0–6.0]	1.0	147.5	0.260	0.277
Tracking indications and limitations	6.0 [5.0–7.0]	5.0 [4.0–6.0]	0.0	146.5	0.117	0.395

**Table 9:** Paired-samples Student’s *t*-test comparison between ELI and TOR.

Questionnaire Item	ELI Mean ± SD	TOR Mean ± SD	$\Delta_{\mathrm{ET}}$ Mean	t(27)	*p*	$d_{z}$
Understanding system state	5.64±1.50	5.43±1.43	0.21	0.86	0.396	0.163
Consistency and predictability	4.93±1.68	4.11±2.01	0.82	2.43	0.022	0.460
Logical interpretation	5.39±1.66	4.96±1.57	0.43	1.20	0.242	0.226
Tracking indications and limitations	5.43±1.60	4.82±1.52	0.61	1.70	0.101	0.321

**Table 10:** Comparison of paired ELI–TOR differences according to Mann–Whitney *U* test.

Questionnaire Item	TOR-First Mean $\Delta_{\mathrm{ET}}$	ELI-First Mean $\Delta_{\mathrm{ET}}$	*U*	*p*
Understanding system state	−0.14	0.57	71.0	0.195
Consistency and predictability	0.93	0.71	109.0	0.623
Logical interpretation	0.29	0.57	95.0	0.907
Tracking indications and limitations	0.64	0.57	96.5	0.963

**Table 11:** Key performance indicators for driving performance.

Category	Key Performance Indicator	Formula
Comfort metrics	Longitudinal Acceleration	$\bar{\left(|\gamma_{x}\left(s\right)|\right)}$
	Longitudinal Jerk	$\bar{\left(|{𝚥}_{x}\left(s\right)|\right)}$
	Lateral Acceleration	$\bar{\left(|\gamma_{y}\left(s\right)|\right)}$
	Lateral Jerk	$\bar{\left(|{𝚥}_{y}\left(s\right)|\right)}$
Safety metrics	Risk of collision	$\bar{RTTCE(s)}+\max\left(RTTCE\left(s\right)\right)$
	Collision events	Number of occurrences
	Out-of-lane events	Number of occurrences

## Data Availability

The data presented in this study are available on request from the corresponding author due to privacy terms agreed with the Institutional Review Board.

## Abbreviations

The following abbreviations are used in this manuscript:

AV	Autonomous Vehicle
HV	Human-Driven Vehicles
TOR	Takeover Request
LLM	Large Language Model
ADS	Autonomous Driving System
AI	Artificial Intelligence
HMI	Human–Machine Interface
LF/HF	Low-Frequency/High-Frequency
UC	Use Case
ECG	Electrocardiography
EDA	Electrodermal Activity
ELI	Empowering Language Interaction
JDM	Joint Decision-Making
LCM	Lightweight Communications and Marshaling
TTS	Text-to-Speech
VAD	Voice Activity Detector
HRV	Heart Rate Variability
STT	Speech-to-Text
SCR	Skin Conductance Response
SCL	Skin Conductance Level
KPI	Key Performance Indicator
RTTCE	Risk from Time to Close Encounter
